# Modeling (not so) rare developmental disorders associated with mutations in the protein-tyrosine phosphatase SHP2

**DOI:** 10.3389/fcell.2022.1046415

**Published:** 2022-11-04

**Authors:** Maja Solman, Daniëlle T. J. Woutersen, Jeroen den Hertog

**Affiliations:** ^1^ Hubrecht Institute-KNAW, University Medical Center Utrecht, Utrecht, Netherlands; ^2^ Institute Biology Leiden, Leiden University, Leiden, Netherlands

**Keywords:** SHP2, Noonan syndrome, Noonan syndrome with multiple lentigenes, metachondromatosis, modeling, fruitfly, zebrafish, mouse

## Abstract

Src homology region 2 (SH2)-containing protein tyrosine phosphatase 2 (SHP2) is a highly conserved protein tyrosine phosphatase (PTP), which is encoded by *PTPN11* and is indispensable during embryonic development. Mutations in *PTPN11* in human patients cause aberrant signaling of SHP2, resulting in multiple rare hereditary diseases, including Noonan Syndrome (NS), Noonan Syndrome with Multiple Lentigines (NSML), Juvenile Myelomonocytic Leukemia (JMML) and Metachondromatosis (MC). Somatic mutations in *PTPN11* have been found to cause cancer. Here, we focus on the role of SHP2 variants in rare diseases and advances in the understanding of its pathogenesis using model systems.

## 1 SHP2 protein, its activation and downstream signaling

**FIGURE 1:**
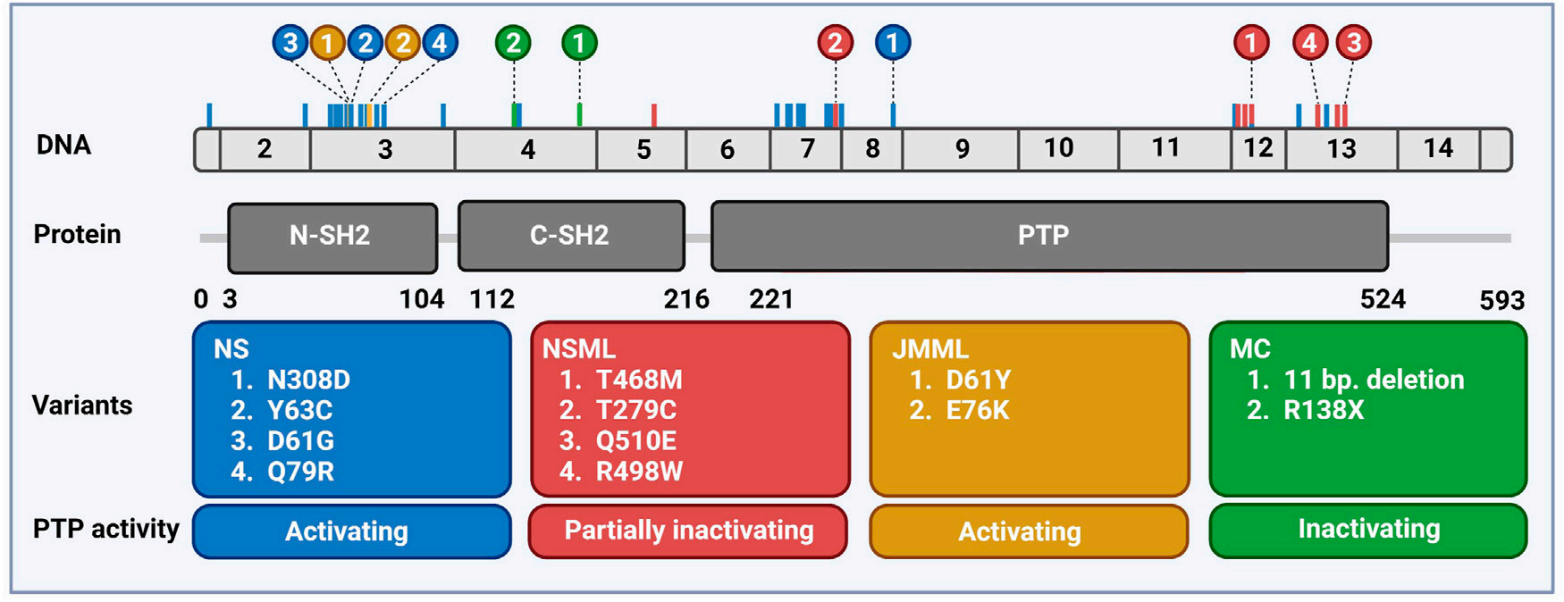
Distribution of PTPN11 genetic variants associated with rare diseases. Schematic overview of the exonic structure of *PTPN11*, consisting of 15 exons (above) and the encoded protein SHP2 (below) with two SH2 domains and the catalytic PTP domain. Frequently occurring mutations are indicated color-coded corresponding to the boxes with the 2–4 most frequently occurring mutations highlighted. Created using BioRender (BioRender.com).

The SH2 domain-containing tyrosine phosphatase 2 (SHP2) is a broadly expressed non-receptor protein tyrosine phosphatase, encoded by the human *PTPN11* gene mapped at chromosomal location 12q24.1 (Freeman et al., 1992; Feng et al., 1993; Vogel et al., 1993). The *PTPN11* gene is comprised of 16 exons producing a 7 kb transcript that contains a 1.779 bp open reading frame which translates into a protein of 593 amino acids (68 kDa) (Grossmann et al., 2010; Tajan et al., 2015a) (Figure 1).

SHP2 protein is built of two tandem SH2 domains (N-SH2 and C-SH2), a catalytic protein tyrosine phosphatase domain (PTP) and a proline rich C-terminal tail all connected *via* flexible polypeptide linkers (Hof et al., 1998). The crystal structure of SHP2 protein (PDB:2SHP) displays a similar structure for N-SH2 and C-SH2 containing a central four-stranded *β*-Sheet enclosing two α-helices. Like all classical PTPs, the structure of the PTP domain includes catalytic loops: P-loop, WPD-loop, K-loop, Q-loop, and pYr-loop, which are involved in substrate binding, formation of the phosphoenzyme complex and release of the phosphate group after the hydrolysis (Barford and Neel, 1998; Hof et al., 1998; Zhang, 2003). The P-loop includes the PTP signature motif [C459(X)_5_R465] enclosing key residues for catalysis. The substrate-enzyme complex is stabilized by the R465 residue. The catalytic cysteine residue, C459, then provokes a nucleophilic attack causing the transfer of the phosphate group from the substrate to C459. The generated phosphocysteinyl moiety is hydrolyzed and the phosphate is released with the help of residues from the WPD loop (N425) and the Q loop (Q506/510) (Barford & Neel, 1998; Hof et al., 1998).

SHP2 activity is regulated through an allosteric mechanism: SHP2 undertakes either a closed conformation, representing the inactive, auto-inhibited state, or an open conformation, representing the active state. The inactive state is established by intra-molecular interaction of the N-SH2 (D’E loop) with the catalytic pocket of the PTP domain, which is then occluded from binding substrates. The C-SH2 domain is not involved in the interface between N-SH2 and PTP, however it supplies binding energy and specificity (Hof et al., 1998). Upon signaling activation SHP2 binds phosphotyrosine motifs on its upstream partners through the N-SH2 and C-SH2 domains through monopartite (only N-SH2 domain) or bipartite phosphotyrosine binding (Marasco et al., 2021). This triggers SHP2 to adopt the open conformational state, releasing the N-SH2 domain from the PTP domain and exposing the catalytic site to its substrates. Distinct SHP2 activators are known, such as receptor tyrosine kinases, insulin receptor substrate 1 (IRS1) and GRB2-associated binding protein 1 (GAB1) (Noguchi et al., 1994; Cunnick et al., 2001; Ekman et al., 2002). Additional mechanisms of SHP2 activation have been proposed. The C-terminal tail of the SHP2 protein includes tyrosine phosphorylation sites Tyr^542^ and Tyr^580^, of which the role is under debate. They are suggested to interact and recruit adaptor proteins, such as GRB2, but have also been proposed to bind SH2 domains of SHP2 and regulate SHP2 activity (Sun et al., 2013).

**FIGURE 2:**
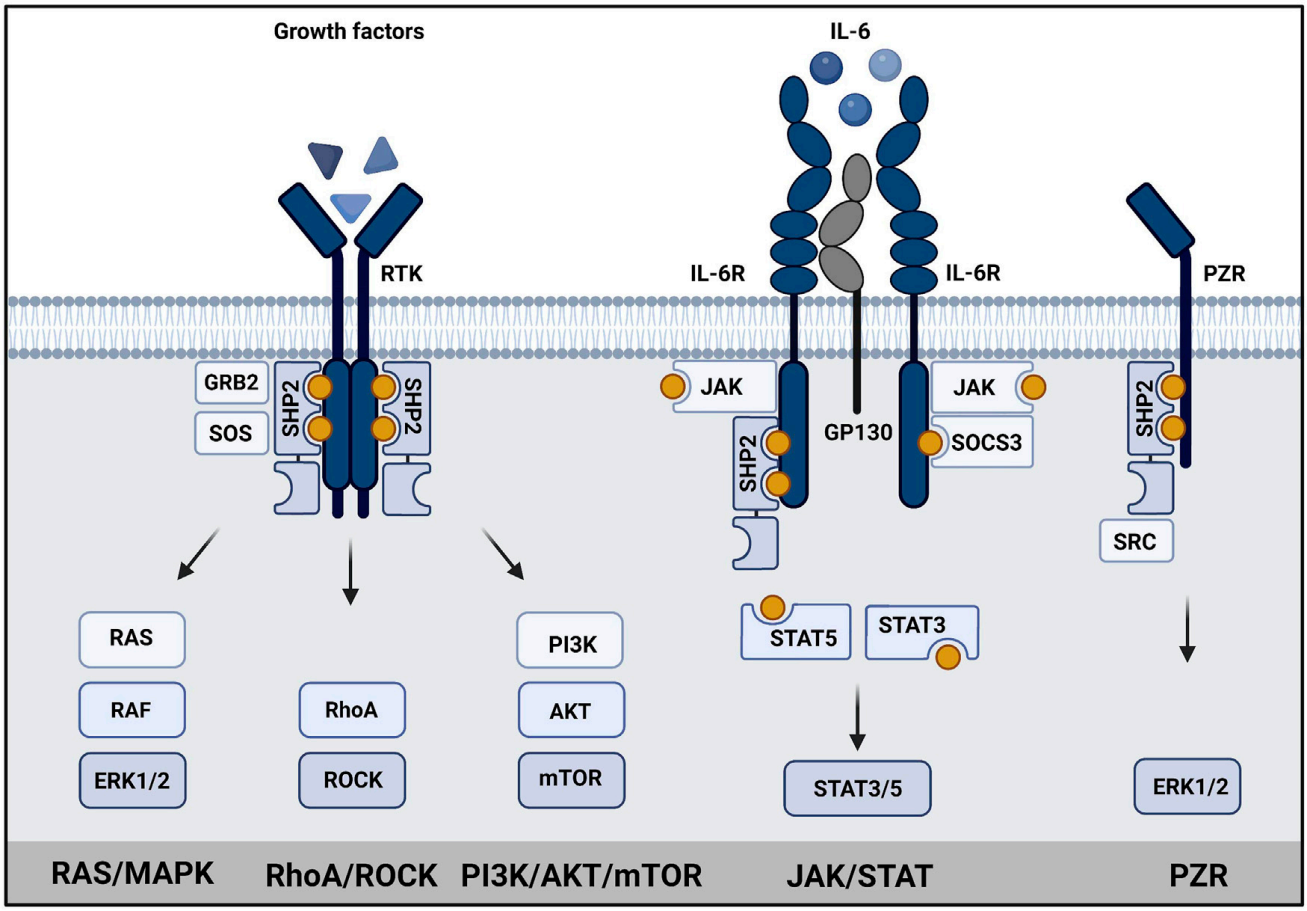
SHP2 has a central role in signaling. Schematic representation of SHP2 signaling in (left) growth factor signaling, (middle) JAK/STAT signaling and (right) PZR signaling. SHP2 binds to phosphotyrosine residues (yellow dots) in transmembrane growth factor receptors, cytokine receptors or PZR *via* its SH2 domains. Subsequently, RAS/MAPK signaling, PI3K/AKT signaling, STAT3/5 signaling or signaling *via* SRC to ERK1/2/MAPK is activated as indicated. Created using BioRender (BioRender.com).

The downstream signaling of SHP2 is rather complex (Figure 2). Upon activation, SHP2 can act on several signaling pathways, mainly promoting signaling. Its role in RAS/MAPK, AKT, and JAK/STAT pathways is best established (Tajan et al., 2015b; Asmamaw et al., 2022). SHP2 signaling is cell type-, developmental stage- and stimulus-dependent. SHP2 is known to dephosphorylate proteins involved in regulation of RAS activity, but may itself act as docking protein as well (Guo & Xu, 2020). RAS activity can be induced by dephosphorylating proteins containing RASA/p120 binding sites, such as GAB1 (Agazie & Hayman, 2003; Montagner et al., 2005; Bard-Chapeau et al., 2006). Dephosphorylation of Sprouty (SPRY) by SHP2 induces release of SPRY from SOS/GRB2 complex, which is then available to activate RAS (Hanafusa et al., 2004; Jarvis et al., 2006). It binds to GAB1/GRB2/SOS1 protein complex as a docking protein at the plasma membrane to facilitate activation of RAS (Bennett et al., 1994; Li et al., 1994). Recruitment of GAB proteins established SHP2’s role in activation of PI3K signaling (Asmamaw et al., 2022). The direct activation of RAS by dephosphorylation of Tyr^32^ on RAS has also been reported (Bunda et al., 2015). An alternative way to activate RAS is through direct or indirect dephosphorylation of the C-terminal Tyr in Src Family Kinases (SFKs), which activates SFKs that finally mediate RAS activation (Ren et al., 2004; Zhang et al., 2004). Recent efforts in understanding SHP2 signaling, such as large scale phospho-proteomic studies led to identification of several putative SHP2 phosphatase targets (Batth et al., 2018; Vemulapalli et al., 2021).

## 2 SHP2 is involved in distinct diseases

### 2.1 SHP2 in cancer

SHP2 is commonly misregulated in cancer either by overexpression or by somatic mutations and has emerged as a prominent cancer drug target. The roles of SHP2 in cancers have been extensively reviewed elsewhere (Zhang et al., 2015; Vainonen et al., 2021). Somatic SHP2 GOF variants are the most common variants (>30%) found in juvenile myelomonocytic leukemia (JMML) (Kratz et al., 2005), a rare but highly aggressive pediatric cancer affecting young children. JMML is characterized by expansion of monocytic and granulocytic blood lineage and hypersensitivity to granulocyte-macrophage colony-stimulating factor (GM-CSF) (Emanuel et al., 1991; Niemeyer & Flotho, 2019). Typical symptoms presented by JMML patients are hepatosplenomegaly, monocytosis, skin rash, lymphadenopathy and respiratory failure. The only treatment is hematopoietic stem cell transplantation (HSCT) with a high relapse rate (50%). SHP2 variants are also present in some other blood cancers, such as myelodysplastic syndrome (10%) and B cell acute lymphoblastic leukemia myelodysplastic syndrome (10%), and seldomly in solid tumors (Kanumuri et al., 2022).

### 2.2 SHP2 in developmental disorders

Given its role in crucial cellular processes, misregulation of SHP2 leads to various pathophysiological conditions. Germline SHP2 variants are found in developmental disorders: GOF mutations are found in 50% of the individuals with Noonan syndrome (NS) and 90% with Noonan syndrome with multiple lentigines (NSML) (Gelb & Tartaglia, 1993; Roberts et al., 2013). NS and NSML together with Costello syndrome, Mazzanti syndrome, Legius syndrome, Neurofibromatosis Type 1, Cardiofaciocutaneous syndrome and CBL (Casitas B-lineage lymphoma) syndrome form the RASopathies group (Tajan et al., 2018a). These syndromes are caused by mutations in components of the RAS/MAPK signaling pathway and share common features. Additionally, germline LOF mutations are found in a rare bone disorder metachondromatosis (MC) (Yang & Neel, 2013).

### 2.3 Noonan syndrome

NS (OMIM 163950) is the most common RASopathy affecting 1:1,000–1:2,500 individuals described for the first time by Jacqueline A. Noonan in 1968 (Noonan, 1968; Van Der Burgt, 2007; Roberts et al., 2013). Noonan syndrome is an autosomal dominant disorder with a vast genetic heterogeneity. In 2001 *PTPN11* variants were discovered as the first causative gene alterations associated to NS (Tartaglia et al., 2001). *PTPN11* variants are present in 50% of NS patients. A growing list of NS-causing variants in other genes exists including *SOS1*, *RAF1*, *RIT1*, *LZTR1*, *KRAS,* and *NRAS,* while for approximately 20% of NS patients the genetic cause is still unclear (El Bouchikhi et al., 2016)*.* The clinical picture of NS patients is heterogeneous, both in regard to a large variety of symptoms and the severity of the disease. Some of the most common features of NS patients are short stature, craniofacial and cardiac defects (Van Der Burgt, 2007; Romano et al., 2010; Zenker et al., 2022). Typical craniofacial defects present in NS are wide-set and down-slanting eyes with palpebral ptosis, low-set posteriorly rotated ears and short webbed neck. These features become less noticeable in adulthood. The short stature is present in 70% of patients, and it has been more prevalent in patients with SHP2 variants (Binder et al., 2005; Grant et al., 2018). Short stature has been associated with growth hormone (GH) insensitivity due to the decreased insulin-like growth factor 1 (IGF-1) levels (Noonan & Kappelgaard, 2015). Cardiac defects are present in 80% of NS patients, of which pulmonary valve stenosis (PVS) is the most common defect (50%–60% of patients), and it is often associated with the occurrence of dysplastic valve and atrium septum defect (ASD) (Grant et al., 2018; Linglart & Gelb, 2020). Hypertrophic cardiomyopathy (HCM) occurs in 20% of NS patients, however it is prevalent in patients with RAF1 variants, whereas SHP2 variants more commonly associate with PVS (Grant et al., 2018; Leoni et al., 2022). Other defects can also be present in NS, such as failure to thrive, chest deformities, including *pectus carinatum* and other skeletal deformities, undescended testicles, delayed puberty, deafness, cutaneous, metabolic, neurocognitive, lymphatic, and bleeding defects (Van Der Burgt, 2007; Yart & Edouard, 2018; Bellio et al., 2019; Gao et al., 2021; Sleutjes et al., 2022). Furthermore, 8-fold higher predisposition to develop cancers has been determined for NS (Kratz et al., 2011). An increased predisposition to develop JMML-like myeloproliferative neoplasm (MPN), a hematological malignancy which rarely progresses to JMML is associated only with specific NS variants of SHP2. Unlike the highly aggressive sporadic JMML, the JMML-like phenotype in NS often intriguingly resolves on its own (Strullu et al., 2014; Niemeyer, 2018).

### 2.4 Noonan syndrome with multiple lentigines

NSML (OMIM 151100) is a dominant disorder formerly known as Leopard Syndrome (LS, Lentigines, Electrocardiographic conduction defects, Ocular hypertelorism, Pulmonary valve stenosis, Abnormalities of genitalia, Retardation of growth and Deafness). NSML is an autosomal dominant disorder, with 85% of patients bearing a *PTPN11* variant, while *BRAF*, *MAP2K1,* or *RAF1* variants are less common. It is a rare disorder, with estimated frequency of 1:3,500. Craniofacial features present in NSML and NS patients overlap, however they occur to be less prominent in NSML patients. Similarly is observed for the short stature, which is common in NS patients, is present in <50% of NSML patients. Cardiac defects are present in 70%–90% of NSML patients but, unlike in NS, HCM is predominant (60%–70%), not PVS, which is common in NS (Grant et al., 2018). Cutaneous features, such as lentigines and “Café-au-lait” spots are common in NSML. Lentigines are present in 90% of cases and are, besides HCM, a cardinal NSML feature. They appear mainly on the hands, neck, face and the upper body and their number increases throughout puberty (Sarkozy et al., 2008). Similarly to NS, NSML patients can present other features, such as chest deformities, delayed puberty, deafness and learning problems (Gelb & Tartaglia, 1993; Sarkozy et al., 2008; Gao et al., 2021).

### 2.5 Metachondromatosis

MC (OMIM 156250) is an autosomal dominant rare bone disorder inherited by incomplete penetrance. MC patients carry heterozygous LOF *PTPN11* variants, including nonsense mutations, frameshifts, splice-site mutations and an 11 base pair deletion (Bowen et al., 2011; Yang & Neel, 2013). During the first decade of life, MC induces benign bone tumors (exostoses) primarily in the hands and feet, as well as enchondromas on long bone metaphyses and iliac crests. Exostoses and enchondromas are usually asymptomatic and they stop occurring after skeletal maturity is reached. MC usually resolves itself spontaneously in adulthood (Yang & Neel, 2013). While heterozygous *Ptpn11* knock-out mouse exhibit no bone deformities, conditional biallelic inactivation of the *Ptpn11* gene within chondrocytes and perichondral cells of heterozygous *Ptpn11* knock-out mice, led to enchondromas in vertebrae and ribs as well as exostoses in the limbs (Kim et al., 2013, 2014; Yang et al., 2013). This suggests that loss of heterozygosity is required for the tumor development, according to Knudson’s two-hit hypothesis. Exostoses observed in the mouse model originate from the *Ptpn11*-null perichondrial cells which fail to differentiate into chondrocytes (Yang et al., 2013; Kim et al., 2014). Whereas the enchondromas derived from *Ptpn11*-null chondrocytes located at the growth plate, seem to originate from unsuccessful transition from cartilage to bone tissue. In the exostoses, decreased pERK1/2 levels were observed (Yang et al., 2013; Kim et al., 2014).

## 3 SHP2 activity is affected in NS and NSML pathogenic variants

Pathogenic SHP2 variants found in NS, NSML, JMML, and MC are largely mutually exclusive for these diseases and the most common variants for each disease are shown in Figure 1. In MC, LOF variants of SHP2 are found (Yang & Neel, 2013), whereas in all three other diseases most of the variants perturb the molecular switch mechanism, inducing a more opened conformation (Yu et al., 2013, 2014; Qiu et al., 2014; LaRochelle et al., 2018; Tao et al., 2021). Based on the NSEuronet database, in NS a large number of pathogenic variants is known to date (>100), with some of them being hot spot variants (e.g., N308D, D61G, Y63C). NS mutations are clustered in exon 3, 8, and 13, and most of them affect the residues involved in the interface between the N-SH2 and the PTP domain (Barford & Neel, 1998). The NS SHP2 variants constitutively adopt the active conformation and display enhanced phosphatase activity, which activates downstream signaling pathways, acting as gain of function (GOF) variants (Keilhack et al., 2005; Kontaridis et al., 2006; Tartaglia et al., 2006). Some variants do not disturb the autoinhibition directly, such as T42A, which has rather drastically increased binding to GAB1, making it more easily activated (Toto et al., 2020).

Several studies addressed whether there is a specific phenotype associated to a specific genotype, however large variations among phenotypes were observed including phenotypical variations among the patients with the same pathogenic SHP2 variant (Tartaglia et al., 2002; Zenker et al., 2004; Shoji et al., 2019). The presence of enhancers and/or suppressors of the phenotype in the genetic background of patients may contribute significantly to the observed phenotypical variation among patients. The only established genotype-phenotype correlation is known for higher predisposition to JMML-like MPN in patients with D61G and T73I variants (Strullu et al., 2014). In sporadic JMML, pathogenic SHP2 variants affect specific residues in exon 3 and 13, increasing the activity of SHP2 in the same way as NS mutations (Tartaglia et al., 2003). However, sporadic JMML variants induce stronger activation of SHP2 than NS variants. It is suggested that the stronger activity of sporadic JMML variants makes them incompatible with embryonic development, which may explain why these mutations are never observed in NS patients (Mason-Suares et al., 2017).

To date, 11 different SHP2 variants have been found in NSML patients, affecting residues in the PTP domain involved in catalysis, leading to a partial loss of the phosphatase activity (Kontaridis et al., 2006; Yu et al., 2013; Qiu et al., 2014). The most common NSML SHP2 variants T468M and Y279C, affect catalysis by provoking rearrangement in the catalytic loops or by inducing loss of substrate affinity, respectively (Qiu et al., 2014). However, upon EGF stimulation of cells, binding to its upstream interacting proteins, such as GAB1, is increased and sustained over time (Kontaridis et al., 2006; Yu et al., 2014). Although NS and NSML SHP2 variants induce opposite effects on the phosphatase activity of SHP2, NS and NSML patients display multiple overlapping phenotypic traits including short stature and facial dysmorphic features observed in NS, although these characteristics are usually milder in NSML patients (Grant et al., 2018). The observed SHP2 activity/patient phenotype discrepancy remains one of the biggest controversies in the field. It was initially suggested that NSML variants act as LOF mutations in the RAS/MAPK signaling pathway upon EGF stimulation in cells (Kontaridis et al., 2006), but also in zebrafish (Stewart et al., 2010) and the hearts of the SHP2-Y279C mouse model (Marin et al., 2011). In contrast, multiple studies reported increased RAS/MAPK signaling in cellular systems (Yu et al., 2013, 2014), fruit-fly (Oishi et al., 2009; Das et al., 2021), zebrafish during gastrulation (Bonetti et al., 2014a), mouse at least in some conditions, such as insulin stimulation (Tajan et al., 2014) and in induced pluripotent stem cells (iPSCs) generated from NSML patients (Carvajal-Vergara et al., 2010). Furthermore, knock-out of one allele of *Ptpn11* has no effects on mouse development, whereas complete knock-out is embryonically lethal (Yang et al., 2006). In addition, the complete LOF SHP2-R465M variant is never found in patients. Based on these findings, it was proposed that activation of RAS/MAPK signaling is established by NSML SHP2 variants, through remaining phosphatase activity and increased binding to its interacting proteins (Yu et al., 2013). Recently, an alternative explanation was proposed, according to which NS and NSML variants have increased liquid-liquid phase separation (LLPS) behavior in common. NS and NSML SHP2 localize to droplets through their well-folded PTP domain. NS and NSML SHP2 variants recruit wild type SHP2 to these droplets as well, which results in strong increases in pERK1/2 levels. Thus, NSML SHP2 variants, despite having reduced PTP activity themselves, promote pERK1/2 signaling through recruitment of wild type SHP2 to the droplets (Zhu et al., 2020). Although many of the NS and NSML symptoms are overlapping, others are specific for the disease and reflect unique mechanisms of NS and NSML SHP2 variant pathogenesis, which could be dependent on the cell type and developmental time window. While both NS and NSML patients develop congenital heart defects, the proportion of patients with a particular heart defect is distinct: in NSML, the majority (85%) of patients presents with HCM (Martínez-Quintana & Rodrguez-González, 2012), whereas in NS PVS is predominant (Tartaglia et al., 2002). Multiple studies suggested that the HCM phenotype is accomplished through activation of the AKT pathway in cardiomyocytes by NSML variants in particular, and its role in HCM pathogenesis was further emphasized by improvement of heart failure in one NSML patient upon treatment with rapamycin (Hahn et al., 2015; Yi et al., 2020). The phenotypes triggered by NS and NSML SHP2 variants can also be completely opposite, as demonstrated by decreased platelet function in NS and increased function in NSML SHP2 variant mice and patients. This was accomplished through opposite effect of NS and NSML variants on the glycoprotein VI (GPVI) ITAM signaling by modulating the phosphorylation of downstream signaling proteins, such as SYK, LAT, AKT, and PLCγ2, which was decreased in NS and increased in NSML platelets (Bellio et al., 2019).

Taken together, more than 100 variants in SHP2 have been found to be associated with different developmental disorders with overlapping and distinct symptoms. Some variants display enhanced catalytic activity and others reduced activity, but catalytic activity by itself does not fully explain the functional differences between the variants. Additional effects of the variants on signaling, which have not been elucidated definitively, must be involved as well.

## 4 Understanding SHP2-variant associated NS and NSML pathogenesis using animal models

**FIGURE 3:**
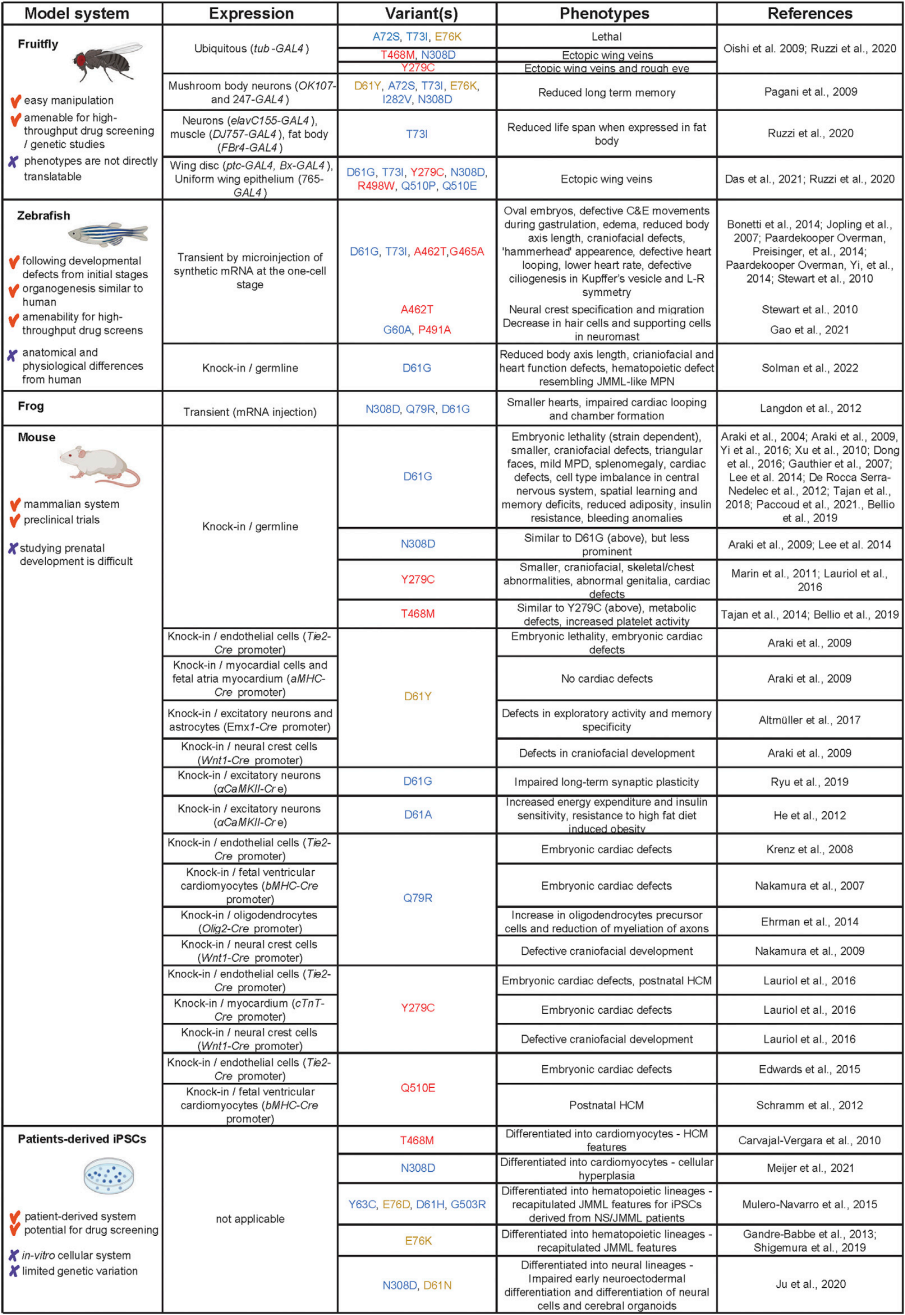
Models used to study SHP2 variants in NS, NSML and leukemia. Advantages and disadvantages of the models are listed in the first column, how the models were derived in the second column. The third column displays the different variants that were used, color-coded as in Figure 1. The observed phenotypes and original references are listed in columns 4 and 5, respectively. Created using BioRender (BioRender.com).

Models used to study pathogenesis of SHP2 variant-associated symptoms range from invertebrate fruitfly and nematodes to vertebrates, including frog, zebrafish and mouse, and patient-derived (iPSCs. Here, we summarize the models that are being used to study the function of SHP2 variants (see Figure 3 for an overview).

### 4.1 Fruit-fly models

Transgenic *Drosophila melanogaster* (fruitfly) expressing the NS, NSML and JMML mutants in corkscrew (*csw*), the fruitfly homologue of *PTPN11*, have been established using the UAS-Gal4 system (Oishi et al., 2006; Pagani et al., 2009; Ruzzi et al., 2020; Das et al., 2021). Whereas fruit-flies ubiquitously expressing NS mutant A72S and JMML mutant E76K are lethal, fruit-flies expressing NSML mutants Y279C and T468M or NS mutant N308D develop ectopic veins on their wings. SHP2 has a central role in RAS/MAPK signaling and since the wing veins in fruitfly emerge at the sites of enhanced RAS/MAPK signaling (Karim & Rubin, 1998), the observed vein phenotype is perhaps not surprising. Fruit-flies expressing the Y279C mutant also develop a rough eye phenotype, another hallmark of enhanced RTK signaling. Both NS and NSML mutants cause the same phenotype through activation of RAS/MAPK signaling, indicated by genetic interaction studies and increased ERK1/2 activation. The wing phenotype depends on phosphatase activity of the mutants, since the Y279C/R465M double mutant containing the phosphatase-dead R465M mutation develops normal wing veins (Oishi et al., 2009). In addition to RAS/MAPK signaling, there is a genetic interaction with the NOTCH, BMP and JAK/STAT signaling pathways (Oishi et al., 2006). Expressing NS and JMML (D61Y, A72S, T73I, E76K, I282V, and N308D), but not NSML (T468M) *csw* variants in mushroom body neurons, which are essential for the formation of memory in fruitfly, leads to reduction in long term memory in response to spaced training. During spaced training, the resting periods between each repetitive training are prolonged. Presumably, mutant *csw* induces elevated ERK1/2 activation during the resting period, which reduces long term memory (Pagani et al., 2009). Another study showed that expression of the T73I variant in the fat body reduces the life span of fruitfly, which may be due to altered metabolism presumably by modulating insulin-like growth factor signaling (Ruzzi et al., 2020). Finally, in an integrated approach using a platform of 13 transgenic fruit-flies expressing human RASopathies variants in *CRAF*, *BRAF*, *KRAS* and *PTPN11* the phenotypic differences by distinct variants were identified, as well as the responses to a wide range of therapeutic treatments and differences in downstream signaling (Das et al., 2021). All *PTPN11* mutant lines induce the formation of ectopic wing veins, specifically cross veins. The pattern of activated signaling pathways is unique for specific *PTPN11* alleles and distinct from the *RAF* and *KRAS* mutants. Activation of the RAS/MAPK pathway is transient for *PTPN11* variants, while the response in the JNK and Hippo pathways is more robust. Similarly, there are large differences in response to different therapeutics between distinct genes and even between distinct variants of the same gene. However, HDAC inhibitors and statins have an effect on most of the transgenic lines.

Although the expression of RASopathies-associated SHP2 variants in fruitfly induces phenotypes that are not directly translatable to NS or NSML in human patients, these studies serve not only as a prominent tool to understand basic pathogenesis principles of RASopathies-associated SHP2 variants, but also to perform drug screens and understand variant-specific phenotypes, downstream signaling differences and drug response, as demonstrated recently for the first time by Das et al. (Das et al., 2021).

### 4.2 Zebrafish and frog models

*Danio rerio* (zebrafish), a complex vertebrate model system with rapid ex-utero embryonic development, represents an exciting system to study defects caused by Shp2 variants during early development. In zebrafish, due to a genome duplication event during evolution, two paralogues exist, *ptpn11a* and *ptpn11b*, of which *ptpn11a* is indispensable for development (Bonetti et al., 2014b). Most of the studies to elucidate the effect of Shp2 variants were performed by injecting synthetic mRNA encoding NS or NSML variants of Shp2a into embryos at the one cell stage, which results in widespread expression during the first 5 days of development. At bud stage at the end of epiboly, zebrafish embryos expressing NS Shp2-D61G or -T73I, and NSML Shp2-A462T or -G465A variants are more oval in shape compared to the round normal embryos. The cell fates do not seem to be affected, while the coordinated convergence and extension (C&E) cell movements during gastrulation are disturbed (Jopling et al., 2007). NS Shp2-D61G and NSML Shp2-A462T variants induce increased Erk1/2 signaling at bud stage and the oval phenotype is rescued using a MEK inhibitor during epiboly (Jopling et al., 2007; Stewart et al., 2010; Bonetti et al., 2014a). Besides Erk1/2 signaling, signaling through Src family kinases, Protein zero related (Pzr), Fer kinases and RhoA are suggested to be involved in the disturbed C&E cell movements during gastrulation (Jopling et al., 2007; Paardekooper Overman et al., 2014a; Paardekooper Overman, Yi, et al., 2014). Later during development, injected embryos are shorter in length, edemic, have craniofacial defects with “hammerhead” appearance and wider set eyes, and display defective cardiac looping (Jopling et al., 2007; Stewart et al., 2010). NS Shp2 variants (G60A and -P491A) decrease the hair cells and supporting cells of the neuromasts (Gao et al., 2021). Expression of NSML Shp2- A462T results in defects in neural crest lineages, including pigmentation defects. The NSML variant expression driven defects in neural crest specification and migration are due to phosphatase and Erk1/2 dependent function of Shp2, whereas the prevention of apoptosis is phosphatase and Erk1/2 independent (Stewart et al., 2010). NS Shp2-D61G and NSML Shp2-A462T variants induce randomized heart displacement in zebrafish embryos, which is associated with overall disruption of left/right asymmetry due to impaired Erk1/2 dependent ciliogenesis and cilia function in Kupffer’s vesicle (Bonetti et al., 2014a).

Expression of Shp2 variants N308D, Q79R or D61G in *Xenopus laevis* embryos leads to the development of smaller hearts. These hearts show impaired cardiac looping and chamber formation as a result of cardiac precursor cell cycle arrest and their inability to incorporate in the developing heart tube. This seems to be due to disruption of the formation of cardiac actin fibers and their polarity, presumably due to defective ROCK signaling (Langdon et al., 2012).

Ectopic expression of Shp2 variants by microinjection is far from ideal: the amount of the Shp2 variant protein varies from embryo to embryo and is not at the physiological level, tissue and developmental stage specific control of expression is lacking and the expression is transient. With the advent of CRISPR gene editing technologies, a genetic zebrafish model carrying the patient-associated single point mutation Shp2-D61G was recently established in our laboratory (Solman et al., 2022). The Shp2-D61G zebrafish are shorter than wild type siblings and have craniofacial and cardiac function defects. Phenotypes are rather mild and both heterozygous and homozygous mutants are viable. In human patients, the SHP2-D61G variant is known to be associated with higher predisposition to JMML (-like MPN)) and clinical evidence suggests that JMML pathogenesis is related to prenatal stages of blood ontogeny (Behnert et al., 2021). The blood ontogeny is conserved in zebrafish. In addition, embryos are transparent and transgenic fluorescent lines are available that allow direct live observations of distinct blood lineages at single cell resolution in developing embryos, which makes the zebrafish an excellent model to study embryonic hematopoiesis (Gore et al., 2018). Mutant Shp2-D61G zebrafish embryos display expansion of the myeloid lineage, which is due to hyperproliferation of hematopoietic stem and progenitor cells (HSPCs). The single cell transcriptomes of HSPCs derived from zebrafish embryos reveal expansion of monocyte/macrophage progenitor cells, which have a striking proinflammatory gene signature (Solman et al., 2022). Transcriptomes of HSPCs from bone marrow of human JMML patients with activating mutations in SHP2, reveal a similar proinflammatory gene expression pattern. Treating zebrafish embryos with the anti-inflammatory drug Dexamethasone reverts the blood phenotype, suggesting anti-inflammatory treatment as an exciting future JMML-(like MPN) drug target at least in JMML patients with activating mutations in SHP2.

Development of zebrafish is a rapid process, which can be followed directly in living embryos. Nonetheless it recapitulates closely human development. NS and NSML Shp2 variant expression in zebrafish induces phenotypes that closely resemble symptoms in NS and NSML patients. Findings derived from the zebrafish model broadens our understanding of the NS and NSML pathogenesis during early development (Jopling et al., 2007; Stewart et al., 2010; Bonetti et al., 2014a; Solman et al., 2022). The technological advances and recent establishment of the first knock-in Shp2-D61G NS zebrafish model (Solman et al., 2022) paves the way for development of further genetic models for distinct SHP2 variants. These will allow to study genotype-phenotype correlations, but also to perform high throughput drug screens.

### 4.3 Mouse models

#### 4.3.1 General phenotypes

Currently, two NS (SHP2-D61G and SHP2-N308D) and two NSML (SHP2-Y279C and SHP2-T468M) knock-in *Mus musculus* (mouse) models expressing SHP2 variants exist (Araki et al., 2004, 2009; Marin et al., 2011; Tajan et al., 2014). Both D61G and N308D mice display typical NS phenotypes: they are smaller in size and weight; have characteristic craniofacial features, such as greater length/width skull ratio, increased inner canthal distance and typical triangular faces; develop mild myeloproliferative disease and splenomegaly after 6 weeks of age; and develop cardiac defects prenatally, including ventricular septal defects (VSD), double-outlet right ventricle (DORV), enlarged valve primordia and thinned myocardium (Araki et al., 2004; Araki et al., 2009). However, the phenotypes in N308D mice are less severe, presumably reflecting the lower level of SHP2 activation of the N308D compared to the D61G allele (Araki et al., 2009). Furthermore, phenotype penetrance is dependent on the mouse strain, likely due to the presence of genetic modifiers. D61G/wt mice show 50% embryonic lethality on the mixed 129S4/SvJae X C57BL6/J background and normal viability on the 129S6/SvEv background. D61G/wt embryos show almost complete lethality on the C57BL/6 background with cardiac defects, edemas, hemorrhages and decreased liver size (Araki et al., 2009). On the C57BL/6NTac X 129S6/SvEvTac background, D61G/wt mice are fully viable and, unlike other genetic backgrounds, developed HCM postnatally with declining cardiac function (Yi et al., 2016).

The Y279C and T468M mice recapitulate features of NSML: they are smaller in size, have abnormal craniofacial characteristics, skeletal/chest abnormalities, mainly *pectus carinatum*, abnormal genitalia, macrophage accumulation in the inner ear, and HCM evident from 12 weeks of age (Marin et al., 2011; Tajan et al., 2014). Hearts from NSML mice are characterized by elevated heart weight to body weight ratio, thickening of the left ventricular wall and septum and increase in cardiomyocyte cell size. With age HCM evolves to dilated cardiomyopathy with decreased cardiac function (Marin et al., 2011; Tajan et al., 2014). In addition to the knock-in NS and NSML mouse models, several mouse models expressing NS and NSML SHP2 variants under distinct promoters were used to study cell-type specific effects, in order to scrutinize the pathogenesis of specific phenotypes.

#### 4.3.2 Cardiac defects

Cardiac defects in D61G and N308D NS mouse models originate in the endothelial cells and they are dependent on ERK1/2 signaling. Araki et al. (2009) demonstrated that expression of the D61Y mutant induced by Tie2-Cre (endothelial cells), and not αMHC-Cre (adult myocardial cells and fetal atria myocardium) or Wnt1-Cre (neural crest), give rise to enlarged endocardial cushions, VSD, DORV and thinned myocardium. This finding is further supported by a model in which the Q79R variant is expressed under the control of Tie2-Cre (Krenz et al., 2008). Valvulogenesis happens during embryonic development when cushion endothelial cells undergo epithelial-mesenchymal transition (EMT) and mesenchymal cells proliferate. Enlarged cushions in the NS mice are formed due to ERK1/2-driven prolonged EMT and mesenchymal proliferation (Krenz et al., 2008; Araki et al., 2009). The increased pERK1/2 seems to be controlled by ErbB family of RTKs, as ErbB family inhibitors decrease the number of mesenchymal cells in the D61G cushion explants. In contrast, a study in which expression of the Q79R mutant is controlled by βMHC-Cre (fetal ventricular cardiomyocytes), pERK1/2 dependent ventricular noncompaction, VSDs and abnormal anatomy of the interventricular groove are observed, suggesting that myocardial cells also contribute to the development of the NS cardiac defects (Nakamura et al., 2007). The NSML mouse SHP2 Y279C knock-in displays phenotypically similar cardiac defects during prenatal development: enlarged valves, septal defects and myocardial wall thinning (Lauriol et al., 2016). Moreover, expression of the Y279C mutant specifically in the endocardium by Tie2-Cre or myocardium by cTnT-Cre, shows the endothelial origin of these defects and partial contribution of myocardium. The effect of Q510E variant expression under the control of Tie2-Cre on the valves confirms this notion, and enhanced EMT underlies the valve enlargement (Edwards et al., 2015). Endothelial expression is required for postnatal development of HCM, which is suggested to be due to perturbing the endocardial-to-myocardial crosstalk during development. Unlike in NS mouse, rather than ERK1/2 activation, AKT signaling is increased and downstream signaling of FOXP1/FGF and NOTCH1/EPHB2 pathways is decreased (Lauriol et al., 2016). However, the expression of Q510E mutation under the control of βMHC, also leads to postnatal development of HCM, which is dependent on AKT/mTOR signaling (Schramm et al., 2012).

Although distinct signaling pathways are recognized to be upregulated, the central role of AKT/mTOR signaling in driving the NSML mice associated HCM is established (Marin et al., 2011; Schramm et al., 2012; Yi et al., 2020). Blocking AKT signaling by the mTOR inhibitor rapamycin or the AKT inhibitor ARQ 092 inhibits the development and progression of HCM in NSML mice, suggesting AKT signaling is an exciting therapeutic target (Marin et al., 2011; Wang et al., 2017). Molecular mechanisms underlying pathways that hyperactivate AKT in NSML-associated HCM still remain poorly understood. The immunoreceptor PZR is hyperphosphorylated in heart lysates of both NS and NSML mice (Paardekooper Overman et al., 2014b). PZR contains two immunoreceptor tyrosine based inhibitory motifs (ITIM), which bind SHP2 once phosphorylated (Zhao & Zhao, 2000). The kinase c-SRC is a probable candidate to phosphorylate PZR, and it also binds more strongly to NS- and NSML- SHP2 mutants. Thus, this leads to enhanced c-SRC, SHP2, and PZR complex formation, and subsequent hyperphosphorylation of PZR and c-SRC activation (Paardekooper Overman et al., 2014b). Knock-out of PZR in the NSML mouse leads to decreased AKT signaling and prevents the development of HCM, indicating a role of PZR in AKT signaling and HCM pathogenesis (Yi et al., 2020). Additionally, c-SRC signals to NF-κB through IKKα phosphorylation and induces IL-6 expression and secretion, which in an autocrine fashion leads to JAK/STAT3 activation and promotion of fibrosis in NSML mouse hearts (Yi et al., 2020). PZR/SHP2 signaling seems to be targetable, since the treatment with low dose of the anticancer drug dasatinib, which is a tyrosine kinase inhibitor, leads to inhibition of PZR hyperphosphorylation and reverses the cardiomyopathy and fibrosis in NSML, as well as HCM in NS mice (Yi et al., 2016; Yi et al., 2021).

#### 4.3.3 Cognitive defects

Cognitive defects are observed in 30%–50% of NS patients. In the SHP2-D61G mouse model, defective SHP2 signaling in brain results in a cell fate shift resulting in imbalance of cell types in central nervous system with more neurons and less astrocytes in the hippocampus and cortex (Gauthier et al., 2007). The SHP2-D61G and, to a lesser extent, SHP2-N308D mouse model display spatial learning and memory deficits, due to enhanced RAS/MAPK signaling, which enhances excitatory synaptic function and impairs hippocampal long-term potentiation (Lee et al., 2014). Related to impaired synaptic plasticity and increased RAS/MAPK signaling, glutamate AMPA and NMDA receptors are differentially expressed during neuronal maturation in SHP2-D61G mouse (Oh et al., 2017). Furthermore, expression of SHP2-D61G in αCaMKII^+^ excitatory, and not vGAT^+^ inhibitory neurons is sufficient to drive the hippocampus-associated memory formation defects and impaired synaptic plasticity (Ryu et al., 2019). The observed cell-type specific effect is caused by higher expression of components of the RAS/MAPK pathway, such as GAB1 and GRB2 in excitatory neurons. Phospho-Y1252 on the GluN2B subunit of NDMAR is a direct substrate of SHP2 and dephosphorylation leads to release of the actin-regulatory adaptor protein NCK2 from the NDMA receptor and hence defective NDMAR mediated neurotransmission in SHP2-D61G mouse (Levy et al., 2018). Expression of SHP2-D61Y in excitatory neurons and astrocytes of the forebrain and ventral pallium under the control of Emx1-Cre induces defects in exploratory activity and memory specificity and impairs surface expression of AMPA and NMDA receptors. Basal level ERK1/2 signaling is increased, but dampened upon neuronal activity, which may be driven by compensatory mechanisms from differential JAK/STAT, PI3K, and mTOR signaling (Altmüller et al., 2017). SHP2 has a critical role in oligodendrocyte differentiation and maturation (Kuo et al., 2010; Liu et al., 2011). Expression of NS-mutant SHP2-Q79R under the control of Olig2-Cre in the ventral telencephalic progenitors and the entire oligodendrocyte lineage leads to a RAS/MAPK signaling-dependent increase in oligodendrocyte precursor cells and a reduction in myelination of axons in the white matter (Ehrman et al., 2014). Expression of JMML-associated GOF mutant SHP2-E76K in neuronal stem cells using Nestin-Cre results in hydrocephalus, due to a decreased number of ependymal cells and abnormal ependymal cilia. Similar defects in ependymal cells, albeit no hydrocephalus, are observed in the NS SHP2-D61G mouse, whereas the NSML SHP2-Y79C mouse does not show such defects (Zheng et al., 2018).

#### 4.3.4 Other anomalies

The characteristic NS craniofacial deformities, including reduced skull lengths, greater inner canthal distances, taller frontal bone distances and reduced mandibular bone length, are induced by expressing SHP2-Q79R and SHP2-D61Y in mouse neural crest under the control of Wnt1-Cre (Araki et al., 2009; Nakamura et al., 2009). The defective craniofacial development is dependent on RAS/MAPK signaling, as treatment of pregnant mutant mice with MEK inhibitor abolishes the observed defects (Nakamura et al., 2009). Short stature in the NS SHP2-D61G mouse model is associated with decreased levels of insulin-like growth factor 1 (IGF-1). The low levels of IGF-1 upon GF stimulation are mediated by increased RAS/MAPK signaling, presumably through increased dephosphorylation of GAB1 in cell lines responsive to GH. Finally, treatment with MEK inhibitor leads to a partial growth increase (De Rocca Serra-Nédélec et al., 2012). Another study demonstrated that the growth plate of the SHP2-D61G mouse is shorter, which is due to a decrease in the length of the hypertrophic zone, which displays impaired chondrocyte differentiation during endochondral ossification. Treatment with MEK inhibitor, but not IGF-1, rescues the growth plate defects, indicating an endogenous effect on endochondral ossification through RAS/MAPK signaling (Tajan et al., 2018b).

SHP2’s involvement in metabolism is complex. The lean phenotype in NSML SHP2-T468M mice is accompanied by reduced adipogenesis, increased energy expenditure and enhanced insulin sensitivity. The adipogenesis and weight are increased in response to a MEK inhibitor, but not to the mTOR inhibitor rapamycin. Furthermore, SHP2-T468M mice are resistant to weight-gain induced by high fat diet (Tajan et al., 2014). When the NS SHP2-D61A mutant is expressed in the forebrain neurons under the control of the CaMKIIα promoter, specifically female mice exhibit resistance to high fat diet-induced obesity. These mice have increased energy expenditure, decreased food intake and improved insulin sensitivity mediated by cross talk of leptin and estrogen signaling (He et al., 2012). A recent study revealed that NS SHP2-D61G mice, although lean with reduced adiposity, are insulin resistant. Insulin resistance is not associated with altered lipid management, but rather with metainflammation of the tissues. The metainflamed state is triggered by macrophages, which have a proinflammatory state induced by SHP2 hyperactivation. Depleting macrophages or inhibiting SHP2 reduces metainflammation and leads to improved insulin sensitivity (Paccoud et al., 2021).

NS patients often suffer from bleeding anomalies. Analysis of the platelet function indicates an opposite effect in NS SHP2-D61G and NSML SHP2-T468M mouse models. Platelets from NS mice show delayed GPVI and C-type lectin-like receptor 2 (CLEC-2) induced aggregation and decreased thrombus growth on a collagen surface under arterial shear stress, whereas platelets from NSML mice show increased activity (Bellio et al., 2019).

#### 4.3.5 Hematopoietic defects

NS patients with specific SHP2 variants have increased predisposition to develop JMML-like MPN, which can in some cases progress to highly aggressive JMML. NS SHP2-D61G mouse develops a mild myeloproliferative disease (MPD) and splenomegaly after 6 weeks of age. The JMML-defining properties, such as myeloproliferation and hypersensitivity to GM-CSF are recapitulated in the mutant mice (Araki et al., 2004; Xu et al., 2010). The HSCs from the SHP2-D61G mouse exhibit defective cycling and have increased short- and long-term repopulating abilities (Xu et al., 2010). They exhibit increased IL3-cytokine induced RAS/MAPK and PI3K signaling. The deletion of GAB2 in the *Ptpn11* ^*D61G/+*^ */Gab2* ^*−/−*^ double mutant mice leads to partially diminished aberrant HSC activity and MPD (Xu et al., 2010). The conditional knock-in models of JMML-associated SHP2 variants E76K or D61Y in hematopoietic lineages reflected the more aggressive JMML phenotype observed in patients (Mohi et al., 2005; Chan et al., 2009; Xu et al., 2011). The inflammatory cytokine profile marrow is known to exist in the JMML patients (Bagby et al., 1988; Freedman et al., 1992; Dong et al., 2016). Although its role in the pathogenesis of JMML has been highlighted both in murine and zebrafish models, the underlying mechanisms remain unclear. A study by Dong et al. proposed that the observed MPD is driven by effects of mutant cells in the hematopoietic niche, in particular mesenchymal stem/progenitor cells and osteoprogenitors, which produce excessive amounts of CC chemokine CCL3 and induce recruitment of monocytes to the niche. Monocytes then hyperactivate HSCs through release of proinflammatory cytokines, such as interleukin 1β (IL-1β) (Dong et al., 2016). However, it is unclear how this is accomplished in sporadic JMML patients, where the niche cells are not mutated. SHP2-E76K expression driven in myeloid lineage by LysM*-*Cre induces overproduction of IL-1β, which then activates the wild-type HSCs (Yan et al., 2022). The study performed by our laboratory in zebrafish indicates that proinflammatory signaling is induced already in the HSPCs during early differentiation of monocyte/macrophage progenitors and suggests that proinflammatory signaling may be an interesting drug target (Solman et al., 2022).

Knock-in and conditional knock-in mouse models have been essential for understanding of pathogenesis of various SHP2 variant associated NS- and NSML-phenotypes and the cells of origin responsible for these phenotypes. Furthermore, understanding of prenatal and postnatal effects of Shp2 variants, effects of genetic background of mouse strains and genotype-phenotype correlations are being examined using these models. Finally, these models are essential in preclinical trials to identify therapeutic leads.

### 4.4 Induced pluripotent stem cells

As human model for NS and NSML, patient derived iPSCs containing SHP2 variants, are used to study cardiac, neuronal and hematopoietic defects (Carvajal-Vergara et al., 2010; Hamada et al., 2020; Li et al., 2019; Meier et al., 2021; Mulero-Navarro et al., 2015; Pearson et al., 2020). The first iPSCs with SHP2 variants were derived more than a decade ago from skin fibroblasts of two NSML patients carrying the SHP2-T468M mutation (Carvajal-Vergara et al., 2010). These NSML iPSCs display increased RAS/MAPK signaling, in that pMEK1 and pEGFR levels are increased as well as the basal level pERK1/2, which is not further induced by bFGF stimulation. When differentiated into cardiomyocytes these cells show a phenotype consistent with cardiac hypertrophy, with larger surface area compared to wt-iPSCs and HES2 cells, increased sarcomere assembly and nuclear localization of NFATc4. In contrast, both cardiac tissue from NS patients with CRAF and SHP2 variants, and iPSCs with SHP2-N308S variant, display cellular hyperplasia, rather than hypertrophy, which is presumably due to the increased cell cycle progression (Meier et al., 2021). However, hypertrophic phenotypes are observed in cardiomyocytes differentiated from NS-derived iPSCs with CRAF mutations (Jaffré et al., 2019; Nakhaei-Rad et al., 2022). The observed discrepancy may be related to the genetic background of the patients and/or the difference in the mutated gene itself: whereas NS-patients with a mutation in the CR2 domain of *CRAF* develop HCM in more than 90% of cases, this is the case for only 10% of NS-patients with *PTPN11* mutations. iPSCs from skin fibroblasts of two NS patients with SHP2-Y63C and E76D variants and two NS/JMML patients with SHP2-D61H and G503R variants show increased and sustained pERK1/2 levels after EGF stimulation compared to wt iPCSs. When differentiated to hematopoietic progenitors and precursor cells, the NS/JMML iPSCs are hypersensitive to GM-CSF and display hyperproliferation of myeloid lineages, recapitulating JMML features. The NS/JMML iPSCs have increased pSTAT5 signaling and elevated miR-223 and -15a expression levels, which is also observed in bone marrow mononuclear cells from 60% of JMML patients. Myeloid lineage expansion of NS/JMML iPSCs is normalized upon inhibition of miR-223’s function (Mulero-Navarro et al., 2015). Proteomic analysis revealed association of TP53 and NF-κB signaling with NS/JMML iPSCs-derived myeloid cells. The NF-κB inhibitor CBL0137 has an effect specifically on clonogenic myeloid cells from JMML patients, but not on cells from healthy individuals (Pearson et al., 2020). iPSCs generated from two JMML patients with somatic SHP2-E76K mutation upon differentiation into the myeloid lineage, show hyperproliferation, constitutive activation of GM-CSF and increased levels of pSTAT5. The GM-CSF independence is ameliorated by MEK inhibition (Gandre-Babbe et al., 2013). iPSCs generated from another JMML patients with SHP2-E76K produce hyperproliferated CD34^+^ cells and CD34^+^CD45^+^ cells during hematopoietic differentiation. Additional mutations in several genes are present, as revealed by whole exome sequencing. However, the isogenic control cells in which SHP2-E76K variant is reverted to wild type by zinc-finger nuclease-mediated homologous recombination show reversion of defective differentiation of CD34^+^ cells and CD34^+^CD45^+^ cells, indicating the essential role of this SHP2 variant in driving the hematopoietic abnormalities (Shigemura et al., 2019).

Differentiation of NS iPSCs into neural lineages differs from wild type at two time points: impaired early neuroectodermal differentiation of embryonic bodies in NS iPSCs, which is mediated by RAS-MAPK, BMP, and TGF-β signaling pathways; defective differentiation of neural cells and cerebral organoids, with preferred gliogenesis, shortened neurites and decreased neural activities in NS iPSCs, which is rescued by SHP2 inhibition (Ju et al., 2020).

The development of iPSC technologies allows us to study a spectrum of variants in cells derived from human patient material. iPSCs can be differentiated in many distinct cell types, allowing us to study pathogenesis in cell type related to the phenotype of interest. Finally, iPSCs have strong potential to be used in drug screening efforts.

## 5 Moving towards therapies

Since the discovery of *PTPN11* mutations as the cause of Noonan syndrome in 2001, a plethora of preclinical research has been performed in order to better define the pathological mechanisms of the disease and establish potential drug targets. SHP2 is an exciting cancer drug target and four classes of inhibitors are known [for recent reviews, see (Vainonen et al., 2021; Kong & Long, 2022; Song et al., 2022)]: orthosteric inhibitors are directed to the catalytic pocket and block phosphatase activity of SHP2, allosteric inhibitors act as a molecular glue and lock SHP2 in the autoinhibited state, PROTAC degraders target SHP2 for E3 mediated degradation, and interaction inhibitors abolish protein-protein interactions of SHP2 with its binding partners. The discovery of allosteric inhibitors by Novartis in 2016 boosted preclinical cancer studies (Chen et al., 2016), and such inhibitors are currently in clinical trials (Vainonen et al., 2021). Unfortunately, allosteric inhibitors are poorly efficient in affecting the activating NS SHP2 variants (LaRochelle et al., 2018). The recent finding that a peptide, which targets the N-terminal SH2 domain of SHP2, binds activating SHP2 mutants with higher affinity than SHP2 wild-type, may pave the way for this type of inhibitor. Administration of this peptide to zebrafish embryos ameliorates the gastrulation defects and mortality in response to overexpression of Shp2-D61G in zebrafish embryos, providing evidence that his approach is feasible in whole organisms (Bobone et al., 2021). Other strategies for direct targeting of pathogenic SHP2 variants may also be used, including strategies using antisense oligonucleotides, which were used to selectively target the cancerogenic KRAS-G12C variant in whole organisms (Ross et al., 2017).

To date, treatment of SHP2-associated NS and NSML targeted the symptoms, rather than SHP2 itself. For instance, RASopathy patients are treated with recombinant human GF (rhGF) to overcome their short stature, although the effectiveness is not completely established. Treatment with rhGH for 1 year or until (near-) adulthood (Dahlgren & Noordam, 2022) leads to increased growth in the short term (Limal et al., 2006), but remains inconclusive in the long term (Noordam et al., 2008). It is noteworthy that the short term effect on patients with *PTPN11* mutations is less prominent than on patients with other RASopathies-associated mutations (Limal et al., 2006). Long term studies show a broad range of height gain with SD varying from 0.6–1.4 (Giacomozzi et al., 2015). The treatment duration and the start of the therapy prior to puberty seem to have an influence on the effectiveness of the therapy (Stagi et al., 2022).

Preclinical research in the NSML mouse indicates the importance of PI3K/AKT/mTOR signaling for the pathogenesis of HCM (Marin et al., 2011; Schramm et al., 2012; Yi et al., 2020). Furthermore, rapamycin or AKT inhibitor ARQ 092 prevent and revert HCM in NSML mice (Marin et al., 2011; Wang et al., 2017). The treatment of one NSML patient with SHP2-Q510E mutation and progressive HCM with the rapamycin analogue everolimus led to improvement, but not reversal of HCM, prior to heart transplantation. An earlier start of the therapy might be beneficial to prevent cardiac remodeling (Hahn et al., 2015).

Accumulating evidence from preclinical studies implies that targeting RAS/MAPK pathway drugs developed for anti-cancer therapies, such as MEK inhibitors or the farnesyltransferase inhibitor statin, may be beneficial in ameliorating distinct mutant-SHP2 associated phenotypes, such as short stature, cardiac, craniofacial and neurocognitive defects (Nakamura et al., 2007; Araki et al., 2009; De Rocca Serra-Nédélec et al., 2012; Bonetti et al., 2014b; Lee et al., 2014; Tajan et al., 2018b; Das et al., 2021). Intriguingly, a low dose of the MEK inhibitor trametinib drastically improved the clinical and cardiac status of two NS patients with HCM due to *RIT1* mutations (Andelfinger et al., 2019). Trametinib treatment in a NS patient with a *SOS1* mutation and associated severe lymphatic defects completely resolved the lymphatic symptoms after 8 weeks of treatment (Dori et al., 2020). Severe respiratory distress related to lymphatic defects and multifocal atrial tachycardia (MAT) present in a NS patient with *SOS1* mutation (Lioncino et al., 2022), as well as MAT present in a NS patient with *RAF1* mutation (Meisner et al., 2021) were resolved upon trametinib treatment.

The 3-hydroxy-3-methyl-glutaryl-coenzyme A reductase (HMG-CoA reductase) inhibitor lovastatin decreases growth plate defects and cognitive deficits in NS SHP2-D61G mice (Tajan et al., 2018b). Cognitive deficits are also decreased in a mouse model for neurofibromatosis 1 in response to lovastatin (Lee et al., 2014; Li et al., 2005). However, in clinical trials lovastatin and simvastatin do not improve the cognitive dysfunction in NF1 patients (Ottenhoff et al., 2020). Simvastatin is being used in an ongoing clinical trial to determine the efficacy on growth and bone defects in NS patients (ClinicalTrials.gov identifier: NCT02713945).

Recent preclinical studies suggest other interesting potential therapies, such as the protein tyrosine kinase inhibitor dasatinib, which reverts HCM in both NS SHP2-D61G and NSML SHP2-Y279C mice (Yi et al., 2016; Yi et al., 2021), the anti-inflammatory corticosteroid Dexamethasone, which ameliorates the JMML-like MPN in NS Shp2-D61G zebrafish model (Solman et al., 2022), and HDAC inhibitors used in fruitfly (Das et al., 2021).

In conclusion, the preclinical and clinical findings clearly indicate the benefits of RAS/MAPK pathway targeting in NS patients with mutations in different genes, although not yet in patients with *PTPN11* variants. Alternative routes to target SHP2-variant induced NS and NSML have been suggested. The development of fruit-fly and zebrafish models, which facilitate whole organism drug screens, may further boost the future drug target discovery. However, translation of potential therapies identified in preclinical models into the clinic through clinical trials remains challenging. Particularly for the RASopathies this is challenging, because of the rarity of RASopathies, broad spectrum and severity of phenotypes, wide range of underlying genotypes and impact of the time when the therapy is initiated.

## 6 Conclusion

Animal models have greatly contributed towards better understanding of SHP2-associated human developmental disorders. Each model has its advantages and disadvantages. Nevertheless, it remains to be determined why different genetic variants in a single gene provoke such a range of distinct human disorders. Genotype-phenotype correlations in animal models are starting to provide answers. Whereas there is no model that accurately recapitulates all symptoms observed in humans, we believe that zebrafish is the model of choice for genotype-phenotype analyses, because many complex and specific traits that are observed in human patients, including cardiovascular defects, hematological malignancies and neural crest anomalies are also observed in zebrafish. Moreover, the zebrafish is amenable for analysis of early development, because the embryos are easily accessible and many transgenic lines are available, facilitating analysis of cell and tissue fates in live embryos. Better insights into the genotype-phenotype correlation may help to define and/or design therapies for patients suffering from the consequences of expression of SHP2 variants.

## References

[B1] Agazie Y. M. Hayman M. J. (2003). Molecular mechanism for a role of SHP2 in epidermal growth factor receptor signaling. Mol. Cell. Biol. 23 (21), 7875–7886. 10.1128/MCB.23.21.7875-7886.2003 14560030 PMC207628

[B2] Altmüller F. Pothula S. Annamneedi A. Nakhei-Rad S. Montenegro-Venegas C. Pina-Fernández E. (2017). Aberrant neuronal activity-induced signaling and gene expression in a mouse model of RASopathy. PLoS Genet. 13 (3), e1006684. 10.1371/JOURNAL.PGEN.1006684 28346493 PMC5386306

[B3] Andelfinger G. Marquis C. Raboisson M. J. Théoret Y. Waldmüller S. Wiegand G. (2019). Hypertrophic cardiomyopathy in noonan syndrome treated by MEK-inhibition. J. Am. Coll. Cardiol. 73 (17), 2237–2239. 10.1016/J.JACC.2019.01.066 31047013 PMC6916648

[B4] Araki T. Chan G. Newbigging S. Morikawa L. Bronson R. Neel B. G. (2009). Noonan syndrome cardiac defects are caused by PTPN11 acting in endocardium to enhance endocardial-mesenchymal transformation. Proc. Natl. Acad. Sci. U. S. A. 106 (12), 4736–4741. 10.1073/pnas.0810053106 19251646 PMC2649209

[B5] Araki T. Mohi M. G. Ismat F. A. Bronson R. T. Williams I. R. Kutok J. L. (2004). Mouse model of Noonan syndrome reveals cell type- and gene dosage-dependent effects of Ptpn11 mutation. Nat. Med. 10 (8), 849–857. 10.1038/nm1084 15273746

[B6] Asmamaw M. D. Shi X.-J. Zhang L.-R. Liu H.-M. Zhang L.-R. (2022). A comprehensive review of SHP2 and its role in cancer. Cell. Oncol. 2022, 729–753. 10.1007/S13402-022-00698-1 PMC12978123 36066752

[B7] Bagby G. C. Dinarello C. A. Neerhout R. C. Ridgway D. McCall E. (1988). Interleukin 1-dependent paracrine granulopoiesis in chronic granulocytic leukemia of the juvenile type. J. Clin. Invest 82 (4), 1430–1436. 10.1172/JCI113748 3262628 PMC442701

[B8] Bard-Chapeau E. A. Yuan J. Droin N. Long S. Zhang E. E. Nguyen T. v. (2006). Concerted functions of Gab1 and Shp2 in liver regeneration and hepatoprotection. Mol. Cell. Biol. 26 (12), 4664–4674. 10.1128/MCB.02253-05 16738330 PMC1489129

[B9] Barford D. Neel B. G. (1998). Revealing mechanisms for SH2 domain mediated regulation of the protein tyrosine phosphatase SHP-2 Structure 3 London, England: 1993, 6(3), 249–254. 10.1016/S0969-2126(98)00027-6 9551546

[B10] Batth T. S. Papetti M. Pfeiffer A. Tollenaere M. A. X. Francavilla C. Olsen J. V. (2018). Large-scale phosphoproteomics reveals shp-2 phosphatase-dependent regulators of pdgf receptor signaling. Cell Rep. 22 (10), 2784–2796. 10.1016/j.celrep.2018.02.038 29514104 PMC7618100

[B11] Behnert A. Meyer J. Parsa J.-Y. Hechmer A. Loh M. L. Olshen A. (2021). Exploring the genetic and epigenetic origins of juvenile myelomonocytic leukemia using newborn screening samples. Leukemia 36, 279–282. 10.1038/s41375-021-01331-0 34183765 PMC8720242

[B12] Bellio M. Garcia C. Edouard T. Voisin S. Neel B. G. Cabou C. (2019). Catalytic dysregulation of SHP2 leading to Noonan syndromes affects platelet signaling and functions. Blood 134 (25), 2304–2317. 10.1182/BLOOD.2019001543 31562133

[B13] Bennett A. M. Tang T. L. Sugimoto S. Walsh C. T. Neel B. G. (1994). Protein-tyrosine-phosphatase SHPTP2 couples platelet-derived growth factor receptor beta to Ras. Proc. Natl. Acad. Sci. U. S. A. 91 (15), 7335–7339. 10.1073/PNAS.91.15.7335 8041791 PMC44394

[B14] Binder G. Neuer K. Ranke M. B. Wittekindt N. E. (2005). PTPN11 mutations are associated with mild growth hormone resistance in individuals with noonan syndrome. J. Clin. Endocrinol. Metab. 90 (9), 5377–5381. 10.1210/JC.2005-0995 15985475

[B15] Bobone S. Pannone L. Biondi B. Solman M. Flex E. Canale V. C. (2021). Targeting oncogenic src homology 2 domain-containing phosphatase 2 (SHP2) by inhibiting its protein-protein interactions. J. Med. Chem. 64 (21), 15973–15990. 10.1021/acs.jmedchem.1c01371 34714648 PMC8591604

[B16] Bonetti M. Overman J. P. Tessadori F. Noë L. E. Bakkers J. den Hertog J. (2014a). Noonan and LEOPARD syndrome Shp2 variants induce heart displacement defects in zebrafish. Development 141 (9), 1961–1970. 10.1242/dev.106310 24718990

[B17] Bonetti M. Rodriguez-Martinez V. Overman J. P. Overvoorde J. Van Eekelen M. Jopling C. (2014b). Distinct and overlapping functions of ptpn11 genes in zebrafish development. PLoS ONE 9 (4), e94884. 10.1371/journal.pone.0094884 24736444 PMC3988099

[B18] Bowen M. E. Boyden E. D. Holm I. A. Campos-Xavier B. Bonafé L. Superti-Furga A. (2011). Loss-of-Function mutations in PTPN11 cause metachondromatosis, but not ollier disease or maffucci syndrome. PLoS Genet. 7 (4), e1002050. 10.1371/JOURNAL.PGEN.1002050 21533187 PMC3077396

[B19] Bunda S. Burrell K. Heir P. Zeng L. Alamsahebpour A. Kano Y. (2015). Inhibition of SHP2-mediated dephosphorylation of Ras suppresses oncogenesis. Nat. Commun. 6, 8859. 10.1038/NCOMMS9859 26617336 PMC4674766

[B20] Carvajal-Vergara X. Sevilla A. Dsouza S. L. Ang Y. S. Schaniel C. Lee D. F. (2010). Patient-specific induced pluripotent stem-cell-derived models of LEOPARD syndrome. Nature 465 (7299), 808–812. 10.1038/NATURE09005 20535210 PMC2885001

[B21] Chan G. Kalaitzidis D. Usenko T. Kutok J. L. Yang W. Mohi M. G. (2009). Leukemogenic Ptpn11 causes fatal myeloproliferative disorder via cell-autonomous effects on multiple stages of hematopoiesis. Blood 113 (18), 4414–4424. 10.1182/blood-2008-10-182626 19179468 PMC2676094

[B22] Chen Y.-N. P. LaMarche M. J. Chan H. M. Fekkes P. Garcia-Fortanet J. Acker M. G. (2016). Allosteric inhibition of SHP2 phosphatase inhibits cancers driven by receptor tyrosine kinases. Nature 535 (7610), 148–152. 10.1038/nature18621 27362227

[B23] Cunnick J. M. Mei L. Doupnik C. A. Wu J. (2001). Phosphotyrosines 627 and 659 of Gab1 constitute a bisphosphoryl tyrosine-based activation motif (BTAM) conferring binding and activation of SHP2. J. Biol. Chem. 276 (26), 24380–24387. 10.1074/JBC.M010275200 11323411

[B24] Dahlgren J. Noordam C. (2022). Growth, endocrine features, and growth hormone treatment in noonan syndrome. J. Clin. Med. 11 (7), 2034. 10.3390/JCM11072034 35407641 PMC8999676

[B25] Das T. K. Gatto J. Mirmira R. Hourizadeh E. Kaufman D. Gelb B. D. (2021). Drosophila RASopathy models identify disease subtype differences and biomarkers of drug efficacy. IScience 24 (4), 102306. 10.1016/J.ISCI.2021.102306 33855281 PMC8026909

[B26] De Rocca Serra-Nédélec A. Edouard T. Tréguer K. Tajan M. Araki T. Dance M. (2012). Noonan syndrome-causing SHP2 mutants inhibit insulin-like growth factor 1 release via growth hormone-induced ERK hyperactivation, which contributes to short stature. Proc. Natl. Acad. Sci. U. S. A. 109 (11), 4257–4262. 10.1073/pnas.1119803109 22371576 PMC3306697

[B27] Dong L. Yu W. M. Zheng H. Loh M. L. Bunting S. T. Pauly M. (2016). Leukaemogenic effects of Ptpn11 activating mutations in the stem cell microenvironment. Nature 539 (7628), 304–308. 10.1038/nature20131 27783593 PMC5317374

[B28] Dori Y. Smith C. Pinto E. Snyder K. March M. E. Hakonarson H. (2020). Severe lymphatic disorder resolved with MEK inhibition in a patient with noonan syndrome and SOS1 mutation. Pediatrics 146 (6), e20200167. 10.1542/PEDS.2020-0167 33219052

[B29] Edwards M. A. Crombie K. Schramm C. Krenz M. (2015). The Q510E mutation in Shp2 perturbs heart valve development by increasing cell migration. J. Appl. Physiology 118 (1), 124–131. 10.1152/japplphysiol.00008.2014 PMC4281644 25359717

[B30] Ehrman L. A. Nardini D. Ehrman S. Rizvi T. A. Gulick J. Krenz M. (2014). The protein tyrosine phosphatase Shp2 is required for the generation of oligodendrocyte progenitor cells and myelination in the mouse telencephalon. J. Neurosci. 34 (10), 3767–3778. 10.1523/JNEUROSCI.3515-13.2014 24599474 PMC3942589

[B31] Ekman S. Kallin A. Engström U. Heldin C. H. Rönnstrand L. (2002). SHP-2 is involved in heterodimer specific loss of phosphorylation of Tyr771 in the PDGF beta-receptor. Oncogene 21 (12), 1870–1875. 10.1038/SJ.ONC.1205210 11896619

[B32] El Bouchikhi I. Belhassan K. Moufid F. Z. Iraqui Houssaini M. Bouguenouch L. Samri I. (2016). Noonan syndrome-causing genes: Molecular update and an assessment of the mutation rate. Int. J. Pediatr. Adolesc. Med. 3 (4), 133–142. 10.1016/J.IJPAM.2016.06.003 30805484 PMC6372459

[B33] Emanuel P. D. Bates L. J. Castleberry R. P. Gualtieri R. J. Zuckerman K. S. (1991). Selective hypersensitivity to granulocyte-macrophage colony-stimulating factor by juvenile chronic myeloid leukemia hematopoietic progenitors. Blood 77 (5), 925–929. 10.1182/blood.v77.5.925.bloodjournal775925 1704804

[B34] Feng G. S. Hui C. C. Pawson T. (1993). Sh2-containing phosphotyrosine phosphatase as a target of protein-tyrosine kinases. Science 259 (5101), 1607–1611. 10.1126/SCIENCE.8096088 8096088

[B35] Freedman M. H. Cohen A. Grunberger T. Bunin N. Luddy R. E. Saunders E. F. (1992). Central role of tumour necrosis factor, GM-CSF, and interleukin 1 in the pathogenesis of juvenile chronic myelogenous leukaemia. Br. J. Haematol. 80 (1), 40–48. 10.1111/j.1365-2141.1992.tb06398.x 1311195

[B36] Freeman R. M. Plutzky J. Neel B. G. (1992). Identification of a human src homology 2-containing protein-tyrosine-phosphatase: A putative homolog of Drosophila corkscrew. Proc. Natl. Acad. Sci. U. S. A. 89 (23), 11239–11243. 10.1073/PNAS.89.23.11239 1280823 PMC50525

[B37] Gandre-Babbe S. Paluru P. Aribeana C. Chou S. T. Bresolin S. Lu L. (2013). Patient-derived induced pluripotent stem cells recapitulate hematopoietic abnormalities of juvenile myelomonocytic leukemia. Blood 121 (24), 4925–4929. 10.1182/BLOOD-2013-01-478412 23620576 PMC3682343

[B38] Gao X. Huang S. S. Qiu S. W. Su Y. Wang W. Q. Xu H. Y. (2021). Congenital sensorineural hearing loss as the initial presentation of PTPN11-associated noonan syndrome with multiple lentigines or noonan syndrome: Clinical features and underlying mechanisms. J. Med. Genet. 58 (7), 465–474. 10.1136/JMEDGENET-2020-106892 32737134

[B39] Gauthier A. S. Furstoss O. Araki T. Chan R. Neel B. G. Kaplan D. R. R. (2007). Control of CNS cell fate decisions by SHP-2 and its dysregulation in noonan syndrome. Neuron 54 (2), 245–262. 10.1016/J.NEURON.2007.03.027 17442246 PMC1900070

[B40] Gelb B. D. Tartaglia M. (1993). Noonan syndrome with multiple lentigines. Seattle, WA: GeneReviews®. Available at: http://www.ncbi.nlm.nih.gov/pubmed/20301557. 20301557

[B41] Giacomozzi C. Deodati A. Shaikh M. G. Ahmed S. F. Cianfarani S. (2015). The impact of growth hormone therapy on adult height in noonan syndrome: A systematic review. Horm. Res. Paediatr. 83 (3), 167–176. 10.1159/000371635 25721697

[B42] Gore A. V. Pillay L. M. Venero Galanternik M. Weinstein B. M. (2018). The zebrafish: A fintastic model for hematopoietic development and disease. Wiley Interdiscip. Rev. Dev. Biol. 7 (3), e312. 10.1002/wdev.312 29436122 PMC6785202

[B43] Grant A. R. Cushman B. J. Cavé H. Dillon M. W. Gelb B. D. Gripp K. W. (2018). Assessing the gene–disease association of 19 genes with the RASopathies using the ClinGen gene curation framework. Hum. Mutat. 39 (11), 1485–1493. 10.1002/HUMU.23624 30311384 PMC6326381

[B44] Grossmann K. S. Rosário M. Birchmeier C. Birchmeier W. (2010). The tyrosine phosphatase Shp2 in development and cancer. Adv. Cancer Res. 106, 53–89. 10.1016/S0065-230X(10)06002-1 20399956

[B45] Guo W. Xu Q. (2020). Phosphatase-independent functions of SHP2 and its regulation by small molecule compounds. J. Pharmacol. Sci. 144 (3), 139–146. 10.1016/J.JPHS.2020.06.002 32921395

[B46] Hahn A. Lauriol J. Thul J. Behnke-Hall K. Logeswaran T. Schänzer A. (2015). Rapidly progressive hypertrophic cardiomyopathy in an infant with noonan syndrome with multiple lentigines: Palliative treatment with a rapamycin analog. Am. J. Med. Genet. A 167A (4), 744–751. 10.1002/ajmg.a.36982 25708222 PMC4598061

[B47] Hamada A. Akagi E. Obayashi F. Yamasaki S. Koizumi K. Ohtaka M. (2020). Induction of Noonan syndrome-specific human-induced pluripotent stem cells under serum-feeder-and integration-free conditions. Vitro Cell. Dev. Biol. Anim. 56 (10), 888–895. 10.1007/S11626-020-00515-9 PMC7723931 33140329

[B48] Hanafusa H. Torii S. Yasunaga T. Matsumoto K. Nishida E. (2004). Shp2, an SH2-containing protein-tyrosine phosphatase, positively regulates receptor tyrosine kinase signaling by dephosphorylating and inactivating the inhibitor Sprouty. J. Biol. Chem. 279 (22), 22992–22995. 10.1074/JBC.M312498200 15031289

[B49] He Z. Zhang S. S. Meng Q. Li S. Zhu H. H. Raquil M.-A. (2012). Shp2 controls female body weight and energy balance by integrating leptin and estrogen signals. Mol. Cell. Biol. 32 (10), 1867–1878. 10.1128/MCB.06712-11 22431513 PMC3347413

[B50] Hof P. Pluskey S. Dhe-Paganon S. Eck M. J. Shoelson S. E. (1998). Crystal structure of the tyrosine phosphatase SHP-2. Cell 92 (4), 441–450. 10.1016/S0092-8674(00)80938-1 9491886

[B51] Jaffré F. Miller C. L. Schänzer A. Evans T. Roberts A. E. Hahn A. (2019). Inducible pluripotent stem cell-derived cardiomyocytes reveal aberrant extracellular regulated kinase 5 and mitogen-activated protein kinase kinase 1/2 signaling concomitantly promote hypertrophic cardiomyopathy in RAF1-associated noonan syndrome. Circulation 140 (3), 207–224. 10.1161/CIRCULATIONAHA.118.037227 31163979 PMC6709678

[B52] Jarvis L. A. Toering S. J. Simon M. A. Krasnow M. A. Smith-Bolton R. K. (2006). Sprouty proteins are *in vivo* targets of Corkscrew/SHP-2 tyrosine phosphatases. Dev. Camb. Engl. 133 (6), 1133–1142. 10.1242/DEV.02255 16481357

[B53] Jopling C. van Geemen D. den Hertog J. (2007). Shp2 knockdown and noonan/LEOPARD mutant Shp2-induced gastrulation defects. PLoS Genet. 3 (12), e225. 10.1371/journal.pgen.0030225 18159945 PMC2151089

[B54] Ju Y. Park J. S. Kim D. Kim B. Lee J. H. Nam Y. (2020). SHP2 mutations induce precocious gliogenesis of Noonan syndrome-derived iPSCs during neural development *in vitro* . Stem Cell Res. Ther. 11 (1), 209–219. 10.1186/s13287-020-01709-4 32493428 PMC7268229

[B55] Kanumuri R. Kumar Pasupuleti S. Burns S. S. Ramdas B. Kapur R. (2022). Targeting SHP2 phosphatase in hematological malignancies. Expert Opin. Ther. Targets 26 (4), 319–332. 10.1080/14728222.2022.206651810.1080/14728222.2022.2066518 35503226 PMC9239432

[B56] Karim F. D. Rubin G. M. (1998). Ectopic expression of activated Ras1 induces hyperplastic growth and increased cell death in Drosophila imaginal tissues. Development 125 (1), 1–9. 10.1242/DEV.125.1.1 9389658

[B57] Keilhack H. David F. S. McGregor M. Cantley L. C. Neel B. G. (2005). Diverse biochemical properties of Shp2 mutants: Implications for disease phenotypes. J. Biol. Chem. 280 (35), 30984–30993. 10.1074/jbc.M504699200 15987685

[B58] Kim H. K. W. Aruwajoye O. Sucato D. Richards B. S. Feng G. S. Chen D. (2013). Induction of SHP2-deficiency in chondrocytes causes severe scoliosis and kyphosis in mice. Spine 38 (21), E1307–E1312. 10.1097/BRS.0B013E3182A3D370 23873233 PMC3864111

[B59] Kim H. K. W. Feng G. S. Chen D. King P. D. Kamiya N. (2014). Targeted disruption of Shp2 in chondrocytes leads to metachondromatosis with multiple cartilaginous protrusions. J. Bone Min. Res. 29 (3), 761–769. 10.1002/JBMR.2062 PMC4081537 23929766

[B60] Kong J. Long Y. Q. (2022). Recent advances in the discovery of protein tyrosine phosphatase SHP2 inhibitors. RSC Med. Chem. 13 (3), 246–257. 10.1039/D1MD00386K 35434626 PMC8942255

[B61] Kontaridis M. I. Swanson K. D. David F. S. Barford D. Neel B. G. (2006). PTPN11 (Shp2) mutations in LEOPARD syndrome have dominant negative, not activating, effects. J. Biol. Chem. 281 (10), 6785–6792. 10.1074/JBC.M513068200 16377799

[B62] Kratz C. P. Niemeyer C. M. Castleberry R. P. Cetin M. Bergsträsser E. Emanuel P. D. (2005). The mutational spectrum of PTPN11 in juvenile myelomonocytic leukemia and Noonan syndrome/myeloproliferative disease. Blood 106 (6), 2183–2185. 10.1182/blood-2005-02-0531 15928039 PMC1895140

[B63] Kratz C. P. Rapisuwon S. Reed H. Hasle H. Rosenberg P. S. (2011). Cancer in noonan, Costello, cardiofaciocutaneous and LEOPARD syndromes. Am. J. Med. Genet. C Semin. Med. Genet. 157 (2), 83–89. 10.1002/ajmg.c.30300 PMC3086183 21500339

[B64] Krenz M. Gulick J. Osinska H. E. Colbert M. C. Molkentin J. D. Robbins J. (2008). Role of ERK1/2 signaling in congenital valve malformations in Noonan syndrome. Proc. Natl. Acad. Sci. U. S. A. 105 (48), 18930–18935. 10.1073/PNAS.0806556105 19017799 PMC2596231

[B65] Kuo E. Park D. K. Tzvetanova I. D. Leiton C. V. Cho B. S. Colognato H. (2010). Tyrosine phosphatases Shp1 and Shp2 have unique and opposing roles in oligodendrocyte development. J. Neurochem. 113 (1), 200–212. 10.1111/J.1471-4159.2010.06596.X 20132481 PMC2907087

[B66] Langdon Y. Tandon P. Paden E. Duddy J. Taylor J. M. Conlon F. L. (2012). SHP-2 acts via ROCK to regulate the cardiac actin cytoskeleton. Development 139 (5), 948–957. 10.1242/dev.067579 22278918 PMC3274356

[B67] LaRochelle J. R. Fodor M. Vemulapalli V. Mohseni M. Wang P. Stams T. (2018). Structural reorganization of SHP2 by oncogenic mutations and implications for oncoprotein resistance to allosteric inhibition. Nat. Commun. 9 (1), 4508. 10.1038/S41467-018-06823-9 30375388 PMC6207684

[B68] Lauriol J. Cabrera J. R. Roy A. Keith K. Hough S. M. Damilano F. (2016). Developmental SHP2 dysfunction underlies cardiac hypertrophy in Noonan syndrome with multiple lentigines. J. Clin. Invest. 126 (8), 2989–3005. 10.1172/JCI80396 27348588 PMC4966304

[B69] Lee Y. S. Ehninger D. Zhou M. Oh J. Y. Kang M. Kwak C. (2014). Mechanism and treatment for learning and memory deficits in mouse models of Noonan syndrome. Nat. Neurosci. 17 (12), 1736–1743. 10.1038/NN.3863 25383899 PMC4716736

[B70] Leoni C. Blandino R. Delogu A. B. De Rosa G. Onesimo R. Verusio V. (2022). Genotype-cardiac phenotype correlations in a large single-center cohort of patients affected by RASopathies: Clinical implications and literature review. Am. J. Med. Genet. A 188 (2), 431–445. 10.1002/AJMG.A.62529 34643321

[B71] Levy A. D. Xiao X. Shaw J. E. Sudarsana Devi S. P. Katrancha S. M. Bennett A. M. (2018). Noonan syndrome-associated SHP2 dephosphorylates GluN2B to regulate NMDA receptor function. Cell Rep. 24 (6), 1523–1535. 10.1016/J.CELREP.2018.07.006 30089263 PMC6234505

[B72] Li R. Baskfield A. Lin Y. Beers J. Zou J. Liu C. (2019). Generation of an induced pluripotent stem cell line (TRNDi003-A) from a Noonan syndrome with multiple lentigines (NSML) patient carrying a p.Q510P mutation in the PTPN11 gene. Stem Cell Res. 34, 101374. 10.1016/J.SCR.2018.101374 30640061 PMC7017387

[B73] Li W. Cui Y. Kushner S. A. Brown R. A. M. Jentsch J. D. Frankland P. W. (2005). The HMG-CoA reductase inhibitor lovastatin reverses the learning and attention deficits in a mouse model of neurofibromatosis type 1. Curr. Biol. 15 (21), 1961–1967. 10.1016/J.CUB.2005.09.043 16271875

[B74] Li W. Nishimura R. Kashishian A. Batzer A. G. Kim W. J. Cooper J. A. (1994). A new function for a phosphotyrosine phosphatase: Linking GRB2-sos to a receptor tyrosine kinase. Mol. Cell. Biol. 14 (1), 509–517. 10.1128/mcb.14.1.509 8264620 PMC358401

[B75] Limal J. M. Parfait B. Cabrol S. Bonnet D. Leheup B. Lyonnet S. (2006). Noonan syndrome: Relationships between genotype, growth, and growth factors. J. Clin. Endocrinol. Metab. 91 (1), 300–306. 10.1210/JC.2005-0983 16263833

[B76] Linglart L. Gelb B. D. (2020). Congenital heart defects in Noonan syndrome: Diagnosis, management, and treatment. Am. J. Med. Genet. C Semin. Med. Genet. 184 (1), 73–80. 10.1002/AJMG.C.31765 32022400 PMC7682536

[B77] Lioncino M. Fusco A. Monda E. Colonna D. Sibilio M. Caiazza M. (2022). Severe lymphatic disorder and multifocal atrial tachycardia treated with trametinib in a patient with noonan syndrome and SOS1 mutation. Genes 13 (9), 1503. 10.3390/GENES13091503 36140671 PMC9498305

[B78] Liu X. Li Y. Zhang Y. Lu Y. Guo W. Liu P. (2011). SHP-2 promotes the maturation of oligodendrocyte precursor cells through Akt and ERK1/2 signaling *in vitro* . Plos One 6 (6), e21058. 10.1371/JOURNAL.PONE.0021058 21701583 PMC3118803

[B79] Marasco M. Kirkpatrick J. Nanna V. Sikorska J. Carlomagno T. (2021). Phosphotyrosine couples peptide binding and SHP2 activation via a dynamic allosteric network. Comput. Struct. Biotechnol. J. 19, 2398–2415. 10.1016/J.CSBJ.2021.04.040 34025932 PMC8113834

[B80] Marin T. M. Keith K. Davies B. Conner D. A. Guha P. Kalaitzidis D. (2011). Rapamycin reverses hypertrophic cardiomyopathy in a mouse model of LEOPARD syndrome-associated PTPN11 mutation. J. Clin. Invest. 121 (3), 1026–1043. 10.1172/JCI44972 21339643 PMC3049377

[B81] Martínez-Quintana E. Rodrguez-González F. (2012). LEOPARD syndrome caused by Tyr279Cys mutation in the PTPN11 gene. Mol. Syndromol. 2 (6), 251–253. 10.1159/000335995 22822385 PMC3362168

[B82] Mason-Suares H. Toledo D. Gekas J. Lafferty K. A. Meeks N. Pacheco M. C. (2017). Juvenile myelomonocytic leukemia-associated variants are associated with neo-natal lethal Noonan syndrome. Eur. J. Hum. Genet. 10, 509–511. 10.1038/ejhg.2016.202 PMC5386422 28098151

[B83] Meier A. B. Raj Murthi S. Rawat H. Toepfer C. N. Santamaria G. Schmid M. (2021). Cell cycle defects underlie childhood-onset cardiomyopathy associated with Noonan syndrome. IScience 25 (1), 103596. 10.1016/J.ISCI.2021.103596 34988410 PMC8704485

[B84] Meisner J. K. Bradley D. J. Russell M. W. (2021). Molecular management of multifocal atrial tachycardia in noonan’s syndrome with MEK1/2 inhibitor trametinib. Circ. Genom. Precis. Med. 14 (5), e003327. 10.1161/CIRCGEN.121.003327 34463117

[B85] Mohi M. G. Williams I. R. Dearolf C. R. Chan G. Kutok J. L. Cohen S. (2005). Prognostic, therapeutic, and mechanistic implications of a mouse model of leukemia evoked by Shp2 (PTPN11) mutations. Cancer Cell 7 (2), 179–191. 10.1016/J.CCR.2005.01.010 15710330

[B86] Montagner A. Yart A. Dance M. Perret B. Salles J. P. Raynal P. (2005). A novel role for Gab1 and SHP2 in epidermal growth factor-induced Ras activation. J. Biol. Chem. 280 (7), 5350–5360. 10.1074/JBC.M410012200 15574420

[B87] Mulero-Navarro S. Sevilla A. Roman A. C. Lee D.-F. D’Souza S. L. Pardo S. (2015). Myeloid dysregulation in a human induced pluripotent stem cell model of PTPN11 -associated juvenile myelomonocytic leukemia. Cell Rep. 13 (3), 504–515. 10.1016/j.celrep.2015.09.019 26456833 PMC4618050

[B88] Nakamura T. Colbert M. Krenz M. Molkentin J. D. Hahn H. S. Dorn G. W. (2007). Mediating ERK1/2 signaling rescues congenital heart defects in a mouse model of Noonan syndrome. J. Clin. Invest. 117 (8), 2123–2132. 10.1172/JCI30756 17641779 PMC1913487

[B89] Nakamura T. Gulick J. Pratt R. Robbins J. (2009). Noonan syndrome is associated with enhanced pERK activity, the repression of which can prevent craniofacial malformations. Proc. Natl. Acad. Sci. U. S. A. 106 (36), 15436–15441. 10.1073/pnas.0903302106 19706403 PMC2741269

[B90] Nakhaei-Rad S. Bazgir F. Dahlmann J. Busley A. V. Buchholzer M. Haghighi F. (2022). Alteration of myocardial structure and function in RAF1-associated Noonan syndrome: Insights from cardiac disease modeling based on patient-derived iPSCs. BioRxiv Prepr. 10.1101/2022.01.22.477319

[B91] Niemeyer C. M. Flotho C. (2019). Juvenile myelomonocytic leukemia: Who’s the driver at the wheel? Blood 133 (10), 1060–1070. 10.1182/blood-2018-11-844688 30670449

[B92] Niemeyer C. M. (2018). JMML genomics and decisions. Hematol. Am. Soc. Hematol. Educ. Program 2018 (1), 307–312. 10.1182/asheducation-2018.1.307 PMC6245977 30504325

[B93] Noguchi T. Matozaki T. Horita K. Fujioka Y. Kasuga M. (1994). Role of SH-PTP2, a protein-tyrosine phosphatase with Src homology 2 domains, in insulin-stimulated Ras activation. Mol. Cell. Biol. 14 (10), 6674–6682. 10.1128/MCB.14.10.6674 7935386 PMC359197

[B94] Noonan J. A. (1968). Hypertelorism with Turner phenotype. A new syndrome with associated congenital heart disease. Am. J. Dis. Child. 116 (4), 373–380. 10.1001/ARCHPEDI.1968.02100020377005 4386970

[B95] Noonan J. A. Kappelgaard A. M. (2015). The efficacy and safety of growth hormone therapy in children with noonan syndrome: A review of the evidence. Horm. Res. Paediatr. 83 (3), 157–166. 10.1159/000369012 25503994

[B96] Noordam C. Peer P. G. M. Francois I. De Schepper J. van den Burgt I. Otten B. J. (2008). Long-term GH treatment improves adult height in children with Noonan syndrome with and without mutations in protein tyrosine phosphatase, non-receptor-type 11. Eur. J. Endocrinol. 159 (3), 203–208. 10.1530/EJE-08-0413 18562489

[B97] Oh J. Y. Rhee S. Silva A. J. Lee Y. S. Kim H. K. (2017). Noonan syndrome-associated SHP2 mutation differentially modulates the expression of postsynaptic receptors according to developmental maturation. Neurosci. Lett. 649, 41–47. 10.1016/J.NEULET.2017.03.036 28366775 PMC6844293

[B98] Oishi K. Gaengel K. Krishnamoorthy S. Kamiya K. Kim I. K. Ying H. (2006). Transgenic Drosophila models of Noonan syndrome causing PTPN11 gain-of-function mutations. Hum. Mol. Genet. 15 (4), 543–553. 10.1093/HMG/DDI471 16399795

[B99] Oishi K. Zhang H. Gault W. J. Wang C. J. Tan C. C. Kim I. K. (2009). Phosphatase-defective LEOPARD syndrome mutations in PTPN11 gene have gain-of-function effects during Drosophila development. Hum. Mol. Genet. 18 (1), 193–201. 10.1093/HMG/DDN336 18849586 PMC2644650

[B100] Ottenhoff M. J. Krab L. C. Elgersma Y. (2020). Considerations for clinical therapeutic development of statins for neurodevelopmental disorders. ENeuro 7 (2), ENEURO.0392–19.2020. 10.1523/ENEURO.0392-19.2020 PMC7070444 32071072

[B101] Paardekooper Overman J. Preisinger C. Prummel K. Bonetti M. Giansanti P. Heck A. (2014a). Phosphoproteomics-mediated identification of Fer kinase as a target of mutant Shp2 in Noonan and LEOPARD syndrome. PloS One 9, e106682. 10.1371/journal.pone.0106682 25184253 PMC4153654

[B102] Paardekooper Overman J. Yi J.-S. Bonetti M. Soulsby M. Preisinger C. Stokes M. P. (2014b). PZR coordinates Shp2 Noonan and LEOPARD syndrome signaling in zebrafish and mice. Mol. Cell. Biol. 34 (15), 2874–2889. 10.1128/MCB.00135-14 24865967 PMC4135572

[B103] Paccoud R. Saint-Laurent C. Piccolo E. Tajan M. Dortignac A. Pereira O. (2021). SHP2 drives inflammation-triggered insulin resistance by reshaping tissue macrophage populations. Sci. Transl. Med. 13 (591), eabe2587. 10.1126/SCITRANSLMED.ABE2587 33910978

[B104] Pagani M. R. Oishi K. Gelb B. D. Zhong Y. (2009). The phosphatase SHP2 regulates the spacing effect for long-term memory induction. Cell 139 (1), 186–198. 10.1016/J.CELL.2009.08.033 19804763 PMC2770243

[B105] Pearson S. Guo B. Pierce A. Azadbakht N. Brazzatti J. A. Patassini S. (2020). Proteomic analysis of an induced pluripotent stem cell model reveals strategies to treat juvenile myelomonocytic leukemia. J. Proteome Res. 19 (1), 194–203. 10.1021/acs.jproteome.9b00495 31657576 PMC6942217

[B106] Qiu W. Wang X. Romanov V. Hutchinson A. Lin A. Ruzanov M. (2014). Structural insights into Noonan/LEOPARD syndrome-related mutants of protein-tyrosine phosphatase SHP2 (PTPN11). BMC Struct. Biol. 14 (1), 10–11. 10.1186/1472-6807-14-10 24628801 PMC4007598

[B107] Ren Y. Meng S. Mei L. Zhao Z. J. Jove R. Wu J. (2004). Roles of Gab1 and SHP2 in paxillin tyrosine dephosphorylation and Src activation in response to epidermal growth factor. J. Biol. Chem. 279 (9), 8497–8505. 10.1074/JBC.M312575200 14665621

[B108] Roberts A. E. Allanson J. E. Tartaglia M. Gelb B. D. (2013). Noonan syndrome. Lancet 381 (9863), 333–342. 10.1016/S0140-6736(12)61023-X 23312968 PMC4267483

[B109] Romano A. A. Allanson J. E. Dahlgren J. Gelb B. D. Hall B. Pierpont M. E. (2010). Noonan syndrome: Clinical features, diagnosis, and management guidelines. Pediatrics 126 (4), 746–759. 10.1542/PEDS.2009-3207 20876176

[B110] Ross S. J. Revenko A. S. Hanson L. L. Ellston R. Staniszewska A. Whalley N. (2017). Targeting KRAS-dependent tumors with AZD4785, a high-affinity therapeutic antisense oligonucleotide inhibitor of KRAS. Sci. Transl. Med. 9 (394), eaal5253. 10.1126/scitranslmed.aal5253 28615361

[B111] Ruzzi L. R. Schilman P. E. San Martin A. Lew S. E. Gelb B. D. Pagani M. R. (2020). The phosphatase CSW controls life span by insulin signaling and metabolism throughout adult life in Drosophila. Front. Genet. 11, 364. 10.3389/fgene.2020.00364 32457793 PMC7221067

[B112] Ryu H. H. Kim T. H. Kim J. W. Kang M. Park P. Kim Y. G. (2019). Excitatory neuron–specific SHP2-ERK signaling network regulates synaptic plasticity and memory. Sci. Signal. 12 (571), eaau5755. 10.1126/SCISIGNAL.AAU5755 30837304 PMC6800025

[B113] Sarkozy A. Digilio M. C. Dallapiccola B. (2008). Leopard syndrome. Orphanet J. Rare Dis. 3 (1), 13. 10.1186/1750-1172-3-13 18505544 PMC2467408

[B114] Schramm C. Fine D. M. Edwards M. A. Reeb A. N. Krenz M. (2012). The PTPN11 loss-of-function mutation Q510E-Shp2 causes hypertrophic cardiomyopathy by dysregulating mTOR signaling. Am. J. Physiology - Heart Circulatory Physiology 302 (1). 10.1152/ajpheart.00665.2011 22058153

[B115] Shigemura T. Matsuda K. Kurata T. Sakashita K. Okuno Y. Muramatsu H. (2019). Essential role of PTPN11 mutation in enhanced haematopoietic differentiation potential of induced pluripotent stem cells of juvenile myelomonocytic leukaemia. Br. J. Haematol. 187 (2), 163–173. 10.1111/BJH.16060 31222725

[B116] Shoji Y. Ida S. Niihori T. Aoki Y. Okamoto N. Etani Y. (2019). Genotype-phenotype correlation analysis in Japanese patients with Noonan syndrome. Endocr. J. 66 (11), 983–994. 10.1507/ENDOCRJ.EJ18-0564 31292302

[B117] Sleutjes J. Kleimeier L. Leenders E. Klein W. Draaisma J. (2022). Lymphatic abnormalities in noonan syndrome spectrum disorders: A systematic review. Mol. Syndromol. 13 (1), 1–11. 10.1159/000517605 35221870 PMC8832235

[B118] Solman M. Blokzijl-Franke S. Piques F. Yan C. Yang Q. Strullu M. (2022). Inflammatory response in hematopoietic stem and progenitor cells triggered by activating SHP2 mutations evokes blood defects. ELife 11, e73040. 10.7554/ELIFE.73040 35535491 PMC9119675

[B119] Song Y. Wang S. Zhao M. Yang X. Yu B. (2022). Strategies targeting protein tyrosine phosphatase SHP2 for cancer therapy. J. Med. Chem. 65 (4), 3066–3079. 10.1021/ACS.JMEDCHEM.1C02008 35157464

[B120] Stagi S. Ferrari V. Ferrari M. Priolo M. Tartaglia M. (2022). Inside the Noonan “universe”: Literature review on growth, GH/IGF axis and rhGH treatment: Facts and concerns. Front. Endocrinol. 13, 951331. 10.3389/FENDO.2022.951331 PMC9434367 36060964

[B121] Stewart R. A. Sanda T. Widlund H. R. Zhu S. Swanson K. D. Hurley A. D. (2010). Phosphatase-dependent and -independent functions of Shp2 in neural crest cells underlie LEOPARD syndrome pathogenesis. Dev. Cell 18, 750–762. 10.1016/j.devcel.2010.03.009 20493809 PMC3035154

[B122] Strullu M. Caye A. Lachenaud J. Cassinat B. Gazal S. Fenneteau O. (2014). Juvenile myelomonocytic leukaemia and Noonan syndrome. J. Med. Genet. 51 (10), 689–697. 10.1136/jmedgenet-2014-102611 25097206

[B123] Sun J. Lu S. Ouyang M. Lin L.-J. Zhuo Y. Liu B. (2013). Antagonism between binding site affinity and conformational dynamics tunes alternative cis-interactions within Shp2. Nat. Commun. 4, 2037. 10.1038/ncomms3037 23792876 PMC3777412

[B124] Tajan M. Batut A. Cadoudal T. Deleruyelle S. Le Gonidec S. Saint Laurent C. (2014). LEOPARD syndrome-associated SHP2 mutation confers leanness and protection from diet-induced obesity. Proc. Natl. Acad. Sci. U. S. A. 111 (42), E4494–E4503. 10.1073/pnas.1406107111 25288766 PMC4210352

[B125] Tajan M. de Rocca Serra A. Valet P. Edouard T. Yart A. (2015a). SHP2 sails from physiology to pathology. Eur. J. Med. Genet. 58 (10), 509–525. 10.1016/j.ejmg.2015.08.005 26341048

[B126] Tajan M. de Rocca Serra A. Valet P. Edouard T. Yart A. (2015b). SHP2 sails from physiology to pathology. Eur. J. Med. Genet. 58 (10), 509–525. 10.1016/j.ejmg.2015.08.005 26341048

[B127] Tajan M. Paccoud R. Branka S. Edouard T. Yart A. (2018a). The RASopathy family: Consequences of germline activation of the RAS/MAPK pathway. Endocr. Rev. 39 (5), 676–700. 10.1210/ER.2017-00232 29924299

[B128] Tajan M. Pernin-Grandjean J. Beton N. Gennero I. Capilla F. Neel B. G. (2018b). Noonan syndrome-causing SHP2 mutants impair ERK-dependent chondrocyte differentiation during endochondral bone growth. Hum. Mol. Genet. 27 (13), 2276–2289. 10.1093/HMG/DDY133 29659837 PMC6005060

[B129] Tao Y. Xie J. Zhong Q. Wang Y. Zhang S. Luo F. (2021). A novel partially open state of SHP2 points to a “multiple gear” regulation mechanism. J. Biol. Chem. 296, 100538. 10.1016/j.jbc.2021.100538 33722610 PMC8054191

[B130] Tartaglia M. Kalidas K. Shaw A. Song X. Musat D. L. van der Burgt I. (2002). PTPN11 mutations in noonan syndrome: Molecular spectrum, genotype-phenotype correlation, and phenotypic heterogeneity. Am. J. Hum. Genet. 70 (6), 1555–1563. 10.1086/340847 11992261 PMC379142

[B131] Tartaglia M. Martinelli S. Stella L. Bocchinfuso G. Flex E. Cordeddu V. (2006). Diversity and functional consequences of germline and somatic PTPN11 mutations in human disease. Am. J. Hum. Genet. 78 (2), 279–290. 10.1086/499925 16358218 PMC1380235

[B132] Tartaglia M. Mehler E. L. Goldberg R. Zampino G. Brunner H. G. Kremer H. (2001). Mutations in PTPN11, encoding the protein tyrosine phosphatase SHP-2, cause Noonan syndrome. Nat. Genet. 29 (4), 465–468. 10.1038/ng772 11704759

[B133] Tartaglia M. Niemeyer C. M. Fragale A. Song X. Buechner J. Jung A. (2003). Somatic mutations in PTPN11 in juvenile myelomonocytic leukemia, myelodysplastic syndromes and acute myeloid leukemia. Nat. Genet. 34 (2), 148–150. 10.1038/ng1156 12717436

[B134] Toto A. Malagrinò F. Visconti L. Troilo F. Gianni S. (2020). Unveiling the molecular basis of the noonan syndrome-causing mutation T42A of SHP2. Int. J. Mol. Sci. 21 (2), E461. 10.3390/IJMS21020461 31936901 PMC7013464

[B135] Vainonen J. P. Momeny M. Westermarck J. (2021). Druggable cancer phosphatases. Sci. Transl. Med. 13 (588), 2967. 10.1126/scitranslmed.abe2967 33827975

[B136] Van Der Burgt I. (2007). Noonan syndrome. Orphanet J. Rare Dis. 2 (1), 4–6. 10.1186/1750-1172-2-4 17222357 PMC1781428

[B137] Vemulapalli V. Chylek L. A. Erickson A. Pfeiffer A. Gabriel K. H. Larochelle J. (2021). Time-resolved phosphoproteomics reveals scaffolding and catalysis-responsive patterns of SHP2-dependent signaling. ELife 10, e64251. 10.7554/ELIFE.64251 33755016 PMC8024022

[B138] Vogel W. Lammers R. Huang J. Ullrich A. (1993). Activation of a phosphotyrosine phosphatase by tyrosine phosphorylation. Science 259 (5101), 1611–1614. 10.1126/SCIENCE.7681217 7681217

[B139] Wang J. Chandrasekhar V. Abbadessa G. Yu Y. Schwartz B. Kontaridis M. I. (2017). *In vivo* efficacy of the AKT inhibitor ARQ 092 in Noonan Syndrome with multiple lentigines-associated hypertrophic cardiomyopathy. PLOS ONE 12 (6), e0178905. 10.1371/JOURNAL.PONE.0178905 28582432 PMC5459472

[B140] Xu D. Liu X. Yu W.-M. Meyerson H. J. Guo C. Gerson S. L. (2011). Non–lineage/stage-restricted effects of a gain-of-function mutation in tyrosine phosphatase Ptpn11 (Shp2) on malignant transformation of hematopoietic cells. J. Exp. Med. 208 (10), 1977–1988. 10.1084/JEM.20110450 21930766 PMC3182060

[B141] Xu D. Wang S. Yu W. M. Chan G. Araki T. Bunting K. D. (2010). A germline gain-of-function mutation in Ptpn11 (Shp-2) phosphatase induces myeloproliferative disease by aberrant activation of hematopoietic stem cells. Blood 116 (18), 3611–3621. 10.1182/blood-2010-01-265652 20651068 PMC2981480

[B142] Yan Y. Dong L. Chen C. Bunting K. D. Li Q. Stieglitz E. (2022). JMML tumor cells disrupt normal hematopoietic stem cells by imposing inflammatory stress through overproduction of IL-1β. Blood Adv. 6 (1), 200–206. 10.1182/BLOODADVANCES.2021005089 34555844 PMC8753218

[B143] Yang W. Klaman L. D. Chen B. Araki T. Harada H. Thomas S. M. (2006). An Shp2/SFK/Ras/Erk signaling pathway controls trophoblast stem cell survival. Dev. Cell 10 (3), 317–327. 10.1016/J.DEVCEL.2006.01.002 16516835

[B144] Yang W. Neel B. G. (2013). From an orphan disease to a generalized molecular mechanism: PTPN11 loss-of-function mutations in the pathogenesis of metachondromatosis. Rare Dis. 1 (1), e26657. 10.4161/RDIS.26657 25003010 PMC3927490

[B145] Yang W. Wang J. Moore D. C. Liang H. Dooner M. Wu Q. (2013). Ptpn11 deletion in a novel progenitor causes metachondromatosis by inducing hedgehog signalling. Nature 499499 (7459), 7459491–7459495. 10.1038/nature12396 PMC4148013 23863940

[B146] Yart A. Edouard T. (2018). Noonan syndrome: An update on growth and development. Curr. Opin. Endocrinol. Diabetes Obes. 25 (1), 67–73. 10.1097/MED.0000000000000380 29120925

[B147] Yi J.-S. Huang Y. Kwaczala A. T. Kuo I. Y. Ehrlich B. E. Campbell S. G. (2016). Low-dose dasatinib rescues cardiac function in Noonan syndrome. JCI Insight 1 (20), e90220. 10.1172/jci.insight.90220 27942593 PMC5135272

[B148] Yi J. S. Perla S. Enyenihi L. Bennett A. M. (2020). Tyrosyl phosphorylation of PZR promotes hypertrophic cardiomyopathy in PTPN11-associated Noonan syndrome with multiple lentigines. JCI Insight 5 (15), 137753. 10.1172/JCI.INSIGHT.137753 32584792 PMC7455087

[B149] Yi J. S. Perla S. Huang Y. Mizuno K. Giordano F. J. Vinks A. A. (2021). Low-dose dasatinib ameliorates hypertrophic cardiomyopathy in noonan syndrome with multiple lentigines. Cardiovasc. Drugs Ther. 36, 589–604. 10.1007/s10557-021-07169-z 33689087 PMC9270274

[B150] Yu Z. H. Xu J. Walls C. D. Chen L. Zhang S. Zhang R. (2013). Structural and mechanistic insights into LEOPARD syndrome-associated SHP2 mutations. J. Biol. Chem. 288, 10472–10482. 10.1074/jbc.M113.450023 23457302 PMC3624429

[B151] Yu Z. H. Zhang R. Y. Walls C. D. Chen L. Zhang S. Wu L. (2014). Molecular basis of gain-of-function LEOPARD syndrome-associated SHP2 mutations. Biochemistry 53 (25), 4136–4151. 10.1021/BI5002695/ASSET/IMAGES/LARGE/BI-2014-002695_0007 24935154 PMC4081049

[B152] Zenker M. Buheitel G. Rauch R. Koenig R. Bosse K. Kress W. (2004). Genotype-phenotype correlations in Noonan syndrome. J. Pediatr. 144 (3), 368–374. 10.1016/j.jpeds.2003.11.032 15001945

[B153] Zenker M. Edouard T. Blair J. C. Cappa M. (2022). Noonan syndrome: Improving recognition and diagnosis. Arch. Dis. Child. 0, 2021–322858. 10.1136/archdischild-2021-322858 PMC9685729 35246453

[B154] Zhang J. Zhang F. Niu R. (2015). Functions of Shp2 in cancer. J. Cell. Mol. Med. 19 (9), 2075–2083. 10.1111/JCMM.12618 26088100 PMC4568912

[B155] Zhang S. Q. Yang W. Kontaridis M. I. Bivona T. G. Wen G. Araki T. (2004). Shp2 regulates SRC family kinase activity and Ras/Erk activation by controlling Csk recruitment. Mol. Cell 13 (3), 341–355. 10.1016/S1097-2765(04)00050-4 14967142

[B156] Zhang Z. Y. (2003). Mechanistic studies on protein tyrosine phosphatases. Prog. Nucleic Acid. Res. Mol. Biol. 73, 171–220. 10.1016/S0079-6603(03)01006-7 12882518

[B157] Zhao R. Zhao Z. J. (2000). Dissecting the interaction of SHP-2 with PZR, an immunoglobulin family protein containing immunoreceptor tyrosine-based inhibitory motifs. J. Biol. Chem. 275 (8), 5453–5459. 10.1074/JBC.275.8.5453 10681522

[B158] Zheng H. Yu W. M. Waclaw R. R. Kontaridis M. I. Neel B. G. Qu C. K. (2018). Gain-of-function mutations in the gene encoding the tyrosine phosphatase SHP2 induce hydrocephalus in a catalytically dependent manner. Sci. Signal. 11 (522), eaao1591. 10.1126/SCISIGNAL.AAO1591 29559584 PMC5915342

[B159] Zhu G. Xie J. Kong W. Xie J. Li Y. Du L. (2020). Phase separation of disease-associated SHP2 mutants underlies MAPK hyperactivation. Cell 183 (2), 490–502. 10.1016/J.CELL.2020.09.002 33002410 PMC7572904

